# Self-Assembled Sodium Dodecyl Sulfate Structures on Mineral Surfaces Following Rapid Solvent Removal

**DOI:** 10.1021/acs.langmuir.5c00640

**Published:** 2025-05-20

**Authors:** Mariana C. Prado, Bernardo R. A. Neves

**Affiliations:** † Departamento de Física, ICEB, 28115 Universidade Federal de Ouro Preto, Rua Quatro, Campus Universitário Morro do Cruzeiro, CEP 35402-136 Ouro Preto, Brazil; ‡ Departamento de Física, ICEx, 28114 Universidade Federal de Minas Gerais, Avenida Antônio Carlos, 6627, CEP 30123-970 Belo Horizonte, Brazil

## Abstract

Sodium dodecyl sulfate (SDS) is a widely used surfactant with applications ranging from detergents to cell lysis and nanomaterial exfoliation. Additionally, SDS can form self-assembled structures on different substrates under specific conditions. While extensive research has explored SDS self-assembly at the liquid–solid interface, less is known about the structures formed at the solid–air interface following solvent removal. In this study, we investigated SDS self-assembled structures on HOPG (highly oriented pyrolytic graphite), talc, and mica substrates using spin-coating and spread-coating methods. Scanning probe microscopy revealed a range of morphologies, including hemicylindrical micelles, lamellar bilayers, and quasi-1D structures, shaped by the interaction between SDS and the substrate surface. On HOPG, hemicylindrical micelles were observed in dilute solutions, whereas lamellar 2D structures, likely bilayer stacks, formed in both dilute and concentrated samples. On talc, lamellar bilayers demonstrated temporal evolution and thermal stability up to 160 °C. Mica samples exhibited quasi-1D structures, 2D bilayers, and thinner lamellar 2D structures, with evidence suggesting the presence of a methyl-terminated monolayer. Thermal annealing tests indicated that quasi-1D structures lost organization at 60 °C, whereas bilayers remained stable up to 150 °C, at least. The results highlight the complexity of SDS self-assembly at the solid–air interface, emphasizing the critical role of local environmental factors. These findings provide insights into surfactant behavior during solvent removal and establish a foundation for further exploration of self-assembled systems under ambient conditions.

## Graphical abstract

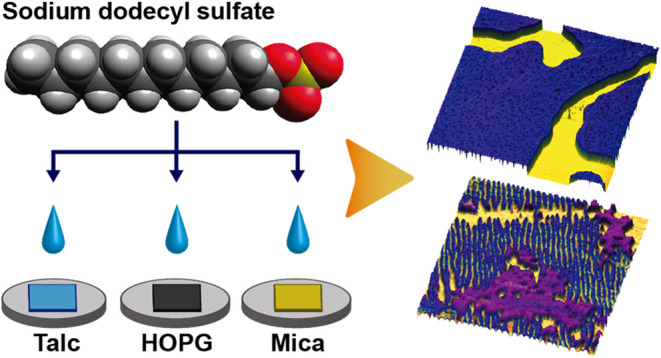

## Introduction

Sodium dodecyl sulfate (SDS) is an organic salt surfactant employed in industry and research for numerous applications. It is used as a detergent in hygiene and household products. It is also useful for lysing cells for nucleic acid extraction and liquid phase exfoliation of nanomaterials.^1,2^ SDS (NaC_12_H_25_SO_4_) is a linear molecule, with 12 carbon atoms in the alkyl chain (hydrophobic tail) and a sulfate group ionically bound to a sodium atom (hydrophilic headgroup). Figure 1a depicts its structure.

**Figure 1:**
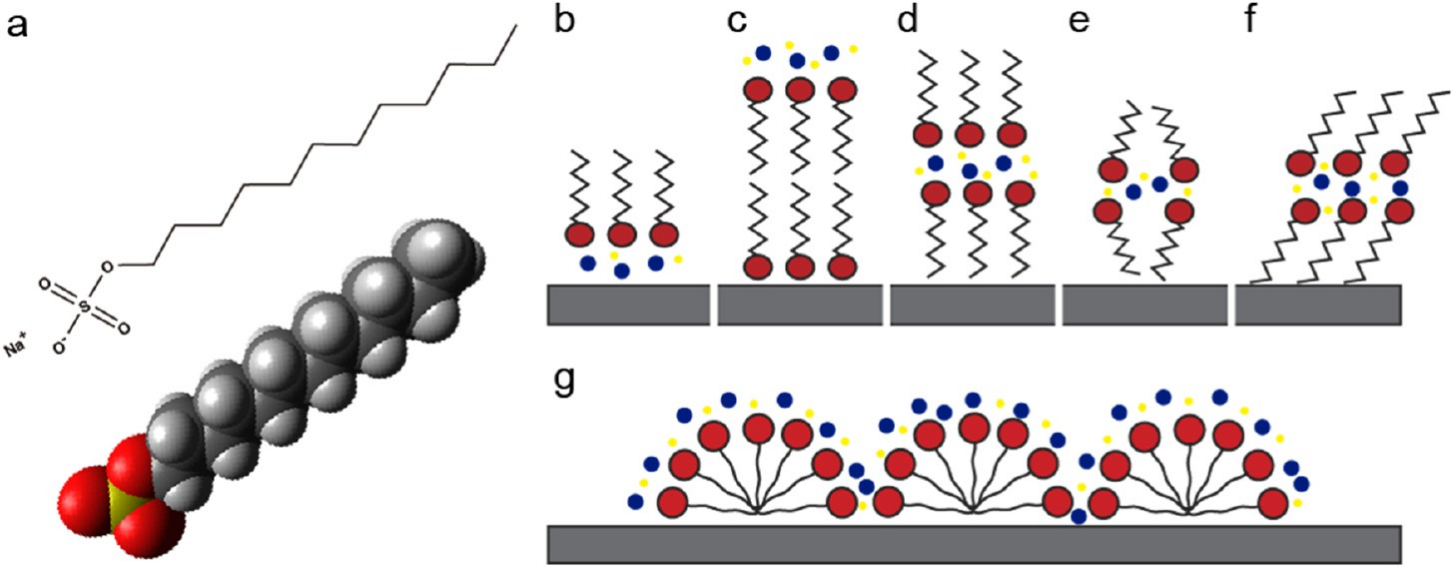
Molecular structure of sodium dodecyl sulfate (SDS – NaC_12_H_25_SO_4_) and schematic representations of possible self-assembled structures. Red circles represent sulfate groups, zigzag lines correspond to alkyl chains, blue circles indicate water molecules, and yellow circles denote Na^+^ ions. (a) SDS molecular structure, with oxygen atoms in red, sulfur in yellow, carbon in dark gray, and hydrogen in light gray. (b) Methyl-terminated monolayer. (c) Sulfate-terminated bilayer with a hydration layer on top. (d) Methyl-terminated bilayer with a hydration layer in the middle. (e) Same as (d), but with less crystalline packing. (f) Methyl-terminated bilayer with tilted alkyl chains and a central hydration layer. (g) Hemicylindrical micelles structures.

Alongside other surfactants, SDS self-assembly structures on various substrates have been extensively studied.^1,3–10^ Figure 1b–g displays schematic representations of possible self-assembled structures SDS could form on a solid interface, depending on the deposition conditions and substrate properties, based on previous studies employing surfactants.^11^ The primary focus was on structures formed at the liquid–solid interface. Scanning probe techniques were employed to image the thin films and tested their mechanical resistance.^7,8^ SDS forms hemicylindrical micelles on HOPG (highly oriented pyrolytic graphite) at the liquid–solid interface (see Figure 1g). This result was first reported by Wanless and Ducker^1,3^ using atomic force microscopy (AFM) and later observations were made employing and force curves to investigate mechanical resistance.^7,8,12^ Jaschke studied the structures formed by surfactants, including SDS, on metallic surfaces.^4^ The stripes formed by SDS hemicylinders on gold displayed fixed angles regarding the substrate steps, unlike those reported on graphite. Levchenko and co-workers^5^ reported the adsorption kinetics of SDS onto substrates functionalized with self-assembled monolayers with both hydrophilic and hydrophobic terminations. Among other conclusions, they observed that the adsorption and desorption rates increase with SDS concentration below the CMC (critical micelle concentration) and vary very little at the CMC and above. They also stated that SDS has a greater affinity for the hydrophobic self-assembled monolayer (SAM). Duan and colleagues^13^ also studied the adsorption of SDS onto a hydrophobic layer using dual polarization interferometry. They found that the critical hemimicelle concentration (HMC) was 1 mM and that the competitive formation of aggregates in solution and at the surface led to different adsorption behaviors at higher concentrations. The time frame of their experiments was on the order of hundreds of seconds, serving as a reference for the time required to reach equilibrium between the solution and the interface. ζ-potential measurements^14^ were also used to study the absorption of SDS onto graphite. The potential’s magnitude increases with an increase in SDS concentration until it reaches a maximum value, indicating the complete formation of micelles on the surface (around the CMC). Works led by Yamada^15,16^ investigated the structure, surface charge distribution, and mechanical properties of SDS self-assembled structures using AFM three-dimensional force mapping. They observed that the surface charge on SDS hemicylinder micelle tops was larger than on trenches between the ripples.

Less attention has been paid to SDS self-assembled structures formed during solvent removal and investigated at the solid–air interface under ambient conditions. The structures formed by SDS on mica upon drop evaporation were investigated using AFM and scanning Kelvin probe microscopy (SKPM) by Bernardes et al.^6,17^ They were interested in the wetting and dewetting dynamics of the SDS–water droplets on mica and reported the formation of self-assembled branched multilayers. The layers, characterized as a lamellar structure (see Figure 1c–f), were hundreds of nanometers thick but exhibited 6 nm steps. These steps were considered a bilayer unit, formed by two SDS molecules and a hydration layer with dissolved Na^+^ ions.

The thick self-assembled multilayers formed by SDS upon solvent evaporation were also studied on borosilicate glass.^18^ Although both mica and the borosilicate glass employed in the studies have a negative charged surface, Shen and Lee^18^ propose a model for SDS adsorption on glass in which a hydration layer forms on the surface and the first layer of SDS molecules adsorb onto that layer with the sulfate group on the external surface and the alkyl chains inside the bilayer. In Bernardes’ model,^17^ the first layer of SDS is adsorbed with the methyl group facing the substrate and the sulfate group in the middle of the bilayer unit.

Forced solvent removal serves as a useful model for understanding surfactant behavior under conditions resembling everyday applications, such as droplet removal of dilute detergents on dishes, skin, or hair.^17^ While SDS self-assembly has been extensively studied at the solid–liquid interface, particularly on HOPG, its behavior at the solid–air interface following rapid solvent removal remains largely unexplored. To the best of our knowledge, no prior studies have systematically investigated the structures formed by SDS on solid substrates after forced solvent removal—such as that induced by spin-coating or spread-coating—using scanning probe microscopy (SPM). This work addresses that gap by examining SDS self-assembly on three mineral surfaces (HOPG, mica, and talc), enabling a comparative analysis of the resulting morphologies and their dependence on substrate properties, solution concentration, and deposition method.

We observed the formation of different types of self-assembled structures, from films and stackings of layers (lamellar structures) to quasi-1D structures (configurations that show a predominant linear or elongated arrangement). Concentrations both under and above CMC were tested, and thin films were observed using scanning probe microscopy. While mica is a negatively charged, highly hydrophilic surface and HOPG is a hydrophobic surface, talc has an intermediary behavior, displaying a neutral cleavage plane.^19^ Although there are water-binding sites on its surface, cohesion forces between water molecules eventually lead to a drop formation and a high contact angle.^20^ Using Bruker’s Peak Force Quantitative Nanomechanical (PF-QNM) mode, comparisons between the SDS film and the substrate adhesion forces were made to determine which part of the molecule (sulfate or methyl group) was on the outer surface of the film. Phase contrast images acquired in tapping mode were also used. Based on our observations, models for the observed structures are devised.

## Experimental Section

Sodium dodecyl sulfate (SDS) was purchased from Thermo Fisher Scientific and used as received. DI (deionized) water (18.2 MΩ·cm resistivity) was used to prepare SDS solutions both below (4 mM) and above (10 and 20 mM) the critical micelle concentration, CMC, (∼8 mM^21^). Solutions were sonicated in a bath at 45 °C to ensure the dissolution of SDS upon making. The substrates were fresh cleaved muscovite mica, HOPG, or talc. It is worth noting that talc substrates were produced by removing small flat sections of the naturally occurring mineral. SDS deposition was accomplished either via spread or spin coating methods. For spread coating deposition, at least 30 s were given for the solution to interact with the substrate before pure N_2_ was used to blow dry the surface.^22^ A homemade spinner was employed as the main deposition method. The substrates were put to spin at moderate speeds (∼4000 rpm) and the SDS solution was dripped on top of it. This method simulated rapid solvent removal akin to forced convection situations that might occur during common handling of detergent solutions in everyday life. Spin coating was the preferable deposition method and spread coating was employed mainly to test if more contact time between solution and substrate would increase coverage or yield a different type of structure.

Scanning probe microscopy (SPM) was used to investigate the self-assembled structures formed by SDS. All images were made with a MultiMode Nanoscope V from Bruker Corporation. Both atomic force microscopy (AFM) tapping mode and PeakForce Quantitative Nanomechanical mapping (PF-QNM) were used to study sample topography. Phase contrast images (tapping) and mechanical properties channels (PF-QNM) were acquired simultaneously with topography to gain insight on sample composition. These signals can be used to help clarify if a given self-assembled surface is formed by the methyl end groups or by the sulfate headgroup, among other properties.^22–25^ To obtain quantitative PF-QNM mapping, the cantilever and tip parameters must first be measured and imputed in the software. Here we perform only qualitative measurements, the contrast indicates more or less adhesive, deformable, or stiff regions, but no numerical value comparison is intended. For the *in situ* annealing studies, a hot-stage AFM setup was employed (Bruker MultiMode SPM with a high-temperature heater accessory). During each experiment, the sample was heated to the target temperature, allowed to reach equilibrium, and subsequently imaged. To prevent the adhesion of sample material caused by the temperature gradient, the AFM tip was also annealed when temperatures exceeded 70 °C.

Commercial silicon cantilevers were used for all measurements. Gwyddion^26^ was used for all image processing. Preliminary investigations of the sample’s temporal evolution are presented in the Supporting Information. Samples were kept in ambient conditions between each analysis.

## Results and Discussion

To ensure clarity in the descriptions that follow, we begin by defining the terminology used to describe the SDS (sodium dodecyl sulfate) structures. The term ’lamella’ refers to fine, planar structures. When these lamellae consist of two molecular layers, they are referred to as bilayers (Figure 1c–f); if composed of a single layer, they are termed monolayers (Figure 1b). Broad, plate-like formations are described as 2D structures (or simply lamellar bilayers or monolayers), while long, narrow formations are referred to as quasi-1D structures, based on their geometry. The term ’hemicylindrical micelles’ is used in accordance with the cited literature to describe structures depicted in Figure 1g.

To investigate SDS self-assembly across mineral surfaces with different properties, we selected three representative substrates: hydrophilic and negatively charged mica, hydrophobic and nonpolar HOPG (highly oriented pyrolytic graphite), and neutral talc, which exhibits intermediate characteristics. Aqueous SDS solutions at three concentrations were employed: 4 mM (below the critical micelle concentration,^21^ CMC) referred to as dilute, 10 mM (slightly above the CMC) as intermediate, and 20 mM (well above the CMC) as concentrated.

We began by examining HOPG, where hemicylindrical micelles at the liquid–solid interface have been widely characterized using scanning probe methods.^1,3,7,8,15,16,27^ Here, we investigated whether similar structures could be detected following solvent removal by spin or spread coating deposition and assisted drying under a nitrogen stream.

Next, our focus shifted to less-explored substrates, namely talc and mica. As phyllosilicate minerals, both materials are of significant practical interest. Clays are an important class of minerals abundant on Earth with many applications in industry and agriculture.^28,29^ Clay powders and detergents are both employed in the cosmetics industry, in many cases, together. These materials can also be exfoliated into two-dimensional platelets using liquid-phase exfoliation methods that employ surfactants.^2,28,30–33^ In this scenario, mineral-surfactant interaction is a key factor to allow exfoliation, promote sheet stabilization, and enable high yield.^30,31^ Mica presents a negatively charged cleavage surface,^34^ whereas talc’s cleavage plane is electrically neutral but retains water-binding sites,^20^ which affects ionic surfactant adsorption and organization.

Finally, we assess the thermal stability of the SDS structures observed on talc and mica by presenting the results of *in situ* annealing experiments, which provide insight into the structural stability and morphological evolution of the self-assembled layers.

### SDS on HOPG

We begin by examining the case of SDS on HOPG. Figure 2 summarizes our findings, SDS hemicylindrical micelles can be detected after water removal. Using the dilute solution and both spin (Figure 2) and spread coating (Figure S1) we observed regions in the HOPG samples covered with stripes, compatible with previous reports. The average periodicity of the stripes measured in the FFT (fast Fourier transform) image was 4.9 and 5.1 nm for the two larger domains seen in the center of Figure 2a. The angle between these domains is 141°. This observation is compatible with previous works and indicates that there is no commensurability between the SDS structures and HOPG’s hexagonal lattice. The thickness of the hemicylinders, 0.4 nm, is less than an SDS molecule length, as reported in earlier works using intermittent contact AFM (atomic force microscopy).^1^ More recent work, employing force modulation, found the hemimicelle radius to be about 2 nm,^15,16^ indicating that the tip–sample forces are high enough to deform the structures under regular intermittent contact measurement conditions, as previously hypothesized. This is important information that will help the interpretation of other results.

**Figure 2:**
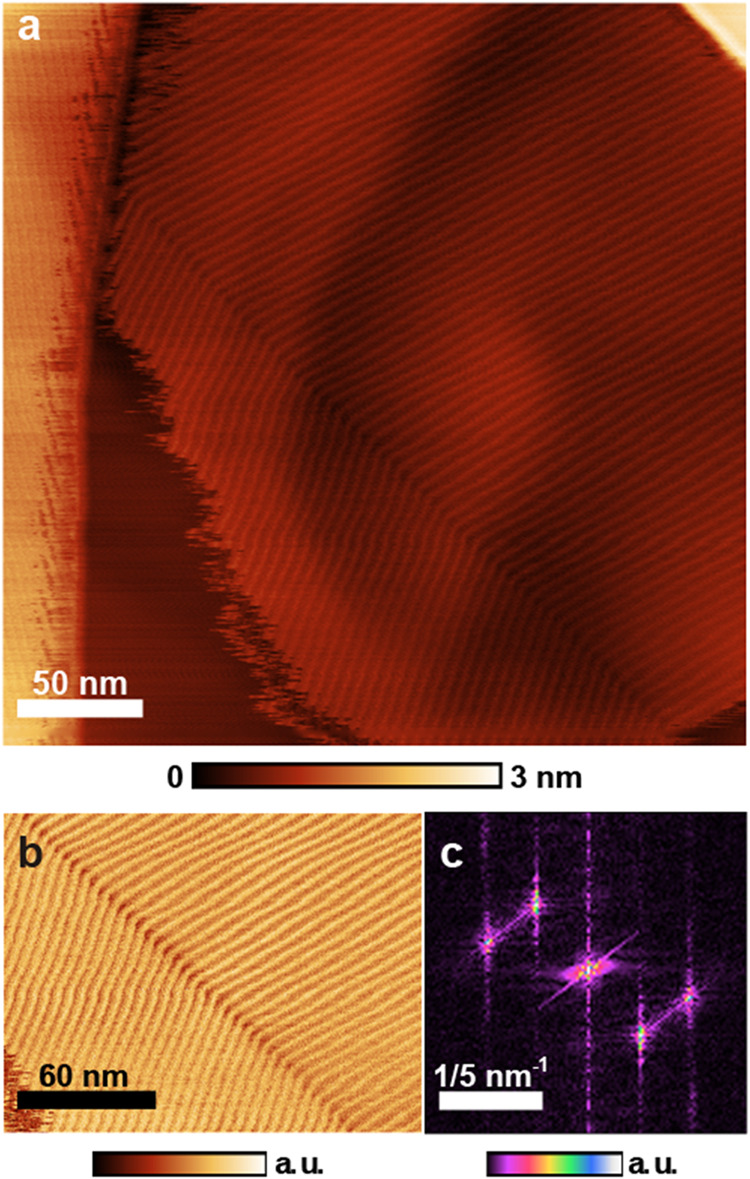
AFM images of SDS spin-coated on HOPG. (a) Topographic tapping mode image showing two domains of hemicylindrical micelles on an HOPG terrace, with an additional domain visible on the higher step at the left side of the image. (b) Phase-contrast image cropped to show only the two larger domains. (c) FFT of the phase image in (b), used to measure periodicity and angle between domains.

In addition, structures consistent with lamellar phases were also detected in these samples. In the Supporting Information (see Figures S1 and S2), a brief description and discussion of these structures are provided. Similar structures were also observed in samples prepared on other substrates, which are discussed in detail later in the text.

### SDS on Talc

We now turn to the case of SDS deposited on talc, a mineral surface with intermediate wetting behavior. As mentioned earlier, it has water-binding sites that make the surface hydrophilic. However, as the number of water molecules on the surface increases, cohesive forces dominate, resulting in the formation of water droplets and the surface behaving hydrophobically.^20^ SDS formed lamellar structures on talc, with coverage and stacking varying with concentration. The solution concentration did not affect the type of structure formed (lamellar). The dilute solution typically yields low substrate coverage and is discussed only in the Supporting Information (see Figure S3).

Figure 3 shows images of a sample produced by spin-coating the 20 mM solution. Panel a displays the topography of the freshly deposited sample, the first time the field was scanned. Three distinct regions can be seen: the substrate (darker) and an SDS lamellar structure of two different thicknesses. The thinner layer (bottom part of the image) has an average thickness of 3.1 nm, and the thicker layer has 3.4 nm. Some even thicker structures can be seen around the holes in the layer; they are less than 1 nm thicker than the surrounding layer. Figure 3b uses color and 3D projection to highlight these structures: the substrate appears in black, the thinner layer in blue, the thicker layer in purple, and the thicker structures in white (which will be discussed in greater detail in the Supporting Information). The 3.1 nm layer exhibits multiple small holes, while the 3.4 nm layer has fewer but larger holes. Figure 3c displays the same region scanned again immediately after capturing the image in panel a. It is possible to observe that the central part of the thinner layer rearranged into the thicker, less-holey structure. Although this rearrangement was detected in other samples, even days after deposition, both types of layers remain, indicating that not all thinner layers will spontaneously reorganize (see Figure S4). The structures in white in Figure 3b are also discussed in the Supporting Information.

**Figure 3:**
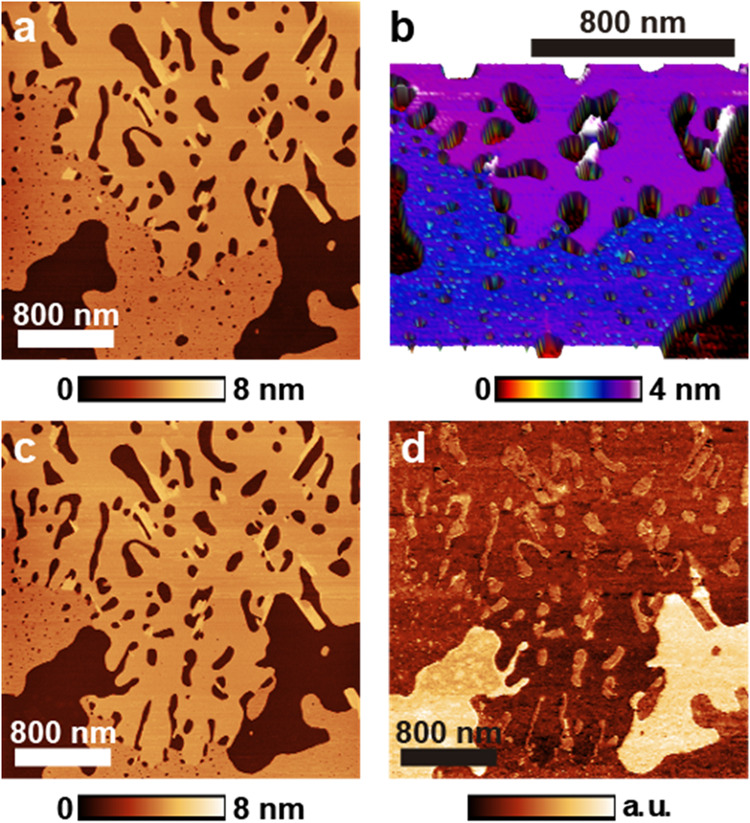
PF-QNM images of the concentrated 20 mM solution spin-coated on talc. (a) Topographic image of a region covered by lamellar structures. (b) 3D projection of the area where the layer height changes. (c) Topographic image of the same region acquired immediately after the panel in (a). It is possible to observe that part of the thinner layer rearranged into the thicker structure. (d) Qualitative adhesion force measurement. Lighter colors indicate greater adhesion force between the tip and sample. SDS structures appear less adhesive.

Panel d shows an adhesive force measurement acquired alongside the topographic image in panel c. It reveals that the substrate is generally more adhesive than the layers. It is notable that both layers have approximately the same adhesion, and that the larger exposed areas of the substrate are much more adhesive than the areas surrounding the holes in the layers. Figure 1, panels c–f, illustrates possible models for the SDS lamellar bilayer structures. Bilayers can vary greatly in thickness depending on the organization of the molecules. Tilted molecules yield thinner layers (Figure 1f). SDS hemihydrate crystals have a lamellar distance of approximately 3.1 nm according to X-ray diffraction measurements^21^ of the monoclinic structure, much less than the value reported by Bernardes^17^ for the SDS bilayer thickness (∼6 nm) on mica. If the alkyl chains of the molecules are interlocked in a quasi-2D crystalline arrangement, the layer will be sturdier and may appear thicker upon AFM imaging. In fact, the cylindrical hemimicelles usually appear less thick than expected (in this work and others, as discussed earlier), likely due to the low organizational state of the alkyl chains. Bearing all this in mind, the lamellar structures in Figure 3 are likely bilayers terminated by the methyl group (low adhesion, further details provided in the SI). The difference in thickness may be due to the differences in alkyl chain organization. The thicker layer does not have small, holey defects within it, indicating that it is probably more crystalline and robust. The tilt of the alkyl chains and the thickness of the water and Na^+^ ion layer may also contribute to the measured thickness of the layer.

Returning to the difference observed in adhesion force on regions of the bare substrate, the substrate is covered by a very thin layer, visible only in the adhesion force channel due to the chosen z-scale for the topographic images (this layer can be more clearly visualized in the images of Figures S3 and S4). It is easily disrupted by the tip during scanning, indicating that it is loosely adhered to the substrate. This layer is believed to be a hydration (or contamination) layer composed of water molecules and ions, due to its high adhesion. The bare regions of the substrate between the thicker SDS layer are less adhesive, probably because less of that contamination layer is present. Further details about the contamination layer and temporal evolution of these samples can be found in the Supporting Information.

Samples produced using the intermediate 10 mM solution also display lamellar structures, as shown in Figure 4. Panel a shows an image of the lamellar layer with an average thickness of 3 nm, similar to the one shown in Figure 3. No regions with the thicker layer were detected near that field. Panel b shows a different sample also produced by spin coating the same solution. The region in this field exhibits a corrugated surface, as shown in greater detail in the inset of Figure 4. The same type of corrugation is observed in samples prepared with the concentrated solution (see Figure S4). The height variation is approximately 1 Å, and the distance between the thinner lines varies between 5 and 16 nm. Tip–sample convolution may hinder further resolution of these structures.

**Figure 4:**
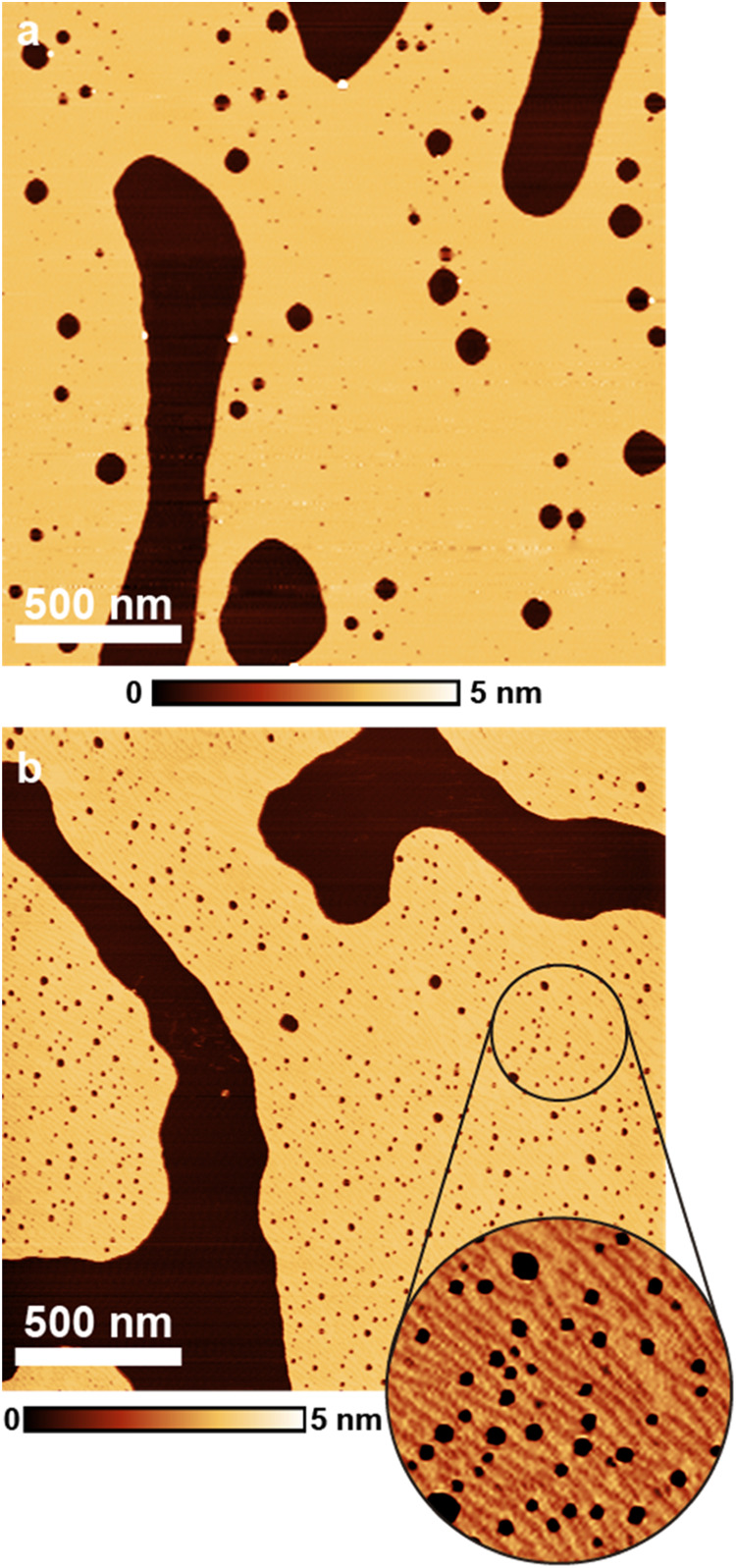
AFM tapping mode images of two different samples made by 10 mM (intermediate) spin-coated on talc. (a) Topographic image of a region covered by a lamellar layer. (b) Topographic image of a region covered by a lamellar layer displaying ripples on its surface. The inset shows a higher magnification of the indicated region.

This could indicate the formation of inverted cylindrical micelles. The local increase in SDS concentration on the surface upon water removal could lead to the formation of inverted micelles, with water molecules, ions, and sulfate groups inside the micelles and the alkyl chains on the outside. A similar observation was made for self-assembled layers of mixed phosphonic acids (mixtures of different alkyl chain length molecules).^22^ Cain and co-workers^35^ reported on self-assembled structures of a wedge-shaped, anionic, surfactant (disodium-3,4,5-tris­(dodecyloxy)­phenylmethylphosphonate – TDPMP) on mica. They reported the formation of multilayered structures that, in high-resolution images, were revealed to consist of periodic stripes. They hypothesized that these stripes were reverse cylindrical TDPMP micelles. Johnson and Nagarajan^36^ proposed a series of possible aggregate structures for cationic surfactants adsorbed on hydrophilic surfaces, including monolayers topped by hemicylinders. These could be somewhat similar to what we observe here (although SDS structures would be reverse micelles, such as reported by Cain et al.^35^).

### SDS on Mica

As mentioned earlier, mica is a phyllosilicate with a negatively charged cleavage plane.^37^ Although the self-assembly of SDS on mica was previously examined,^6,17,18^ the deposition conditions used here yield new and interesting results. Since the dilute (4 mM) solution did not produce complex structures on mica (see the Supporting Information for a brief discussion and image), we proceeded to analyze samples produced with the two solutions with concentrations above the CMC (10 and 20 mM).

Both 10 mM and 20 mM solutions, spread or spin-coated onto mica, yield a variety of self-assembled structures. Samples are highly inhomogeneous, displaying regions covered with different types of structures. Neither concentration nor deposition method variations demonstrated a strong enough influence on the structures to allow control over sample morphology, evidencing that the conditions were subject to intense local variation. No signs of temporal evolution were observed.

Figure 5 summarizes the results. Panel a shows a PF-QNM (peak force quantitative nanomechanical) topographic image of a sample deposited using the concentrated solution spin-coated onto a freshly cleaved substrate. The bare mica substrate appears in dark red, and an SDS multilayered structure is visible on the right side. The first layer (in yellow) is 4.2 nm thick. The subsequent layers are 3.7 nm thick (green, blue, and purple), as shown in the profile presented in panel c. The adhesion force channel, shown in Figure 5b, indicates that the layers are less adhesive (darker) than mica, suggesting that their surface is formed by the SDS methyl group. Panels d and e show topographic and adhesion force channels of another region of the same sample, covered by long, linear structures among much thinner islands of layered material. The linear structures are branched and only a few angstroms thick, varying in width but averaging about 38 nm, while the layers are 1 nm thick. The adhesion force image shows that the substrate appears lighter, thus has the highest adhesion. The branched linear structures have intermediate, and the islands have less adhesion force. Panel f shows an image of a sample spin-coated using the 10 mM solution, demonstrating that the structures are microns long and appear in samples made from both solutions above the CMC.

**Figure 5:**
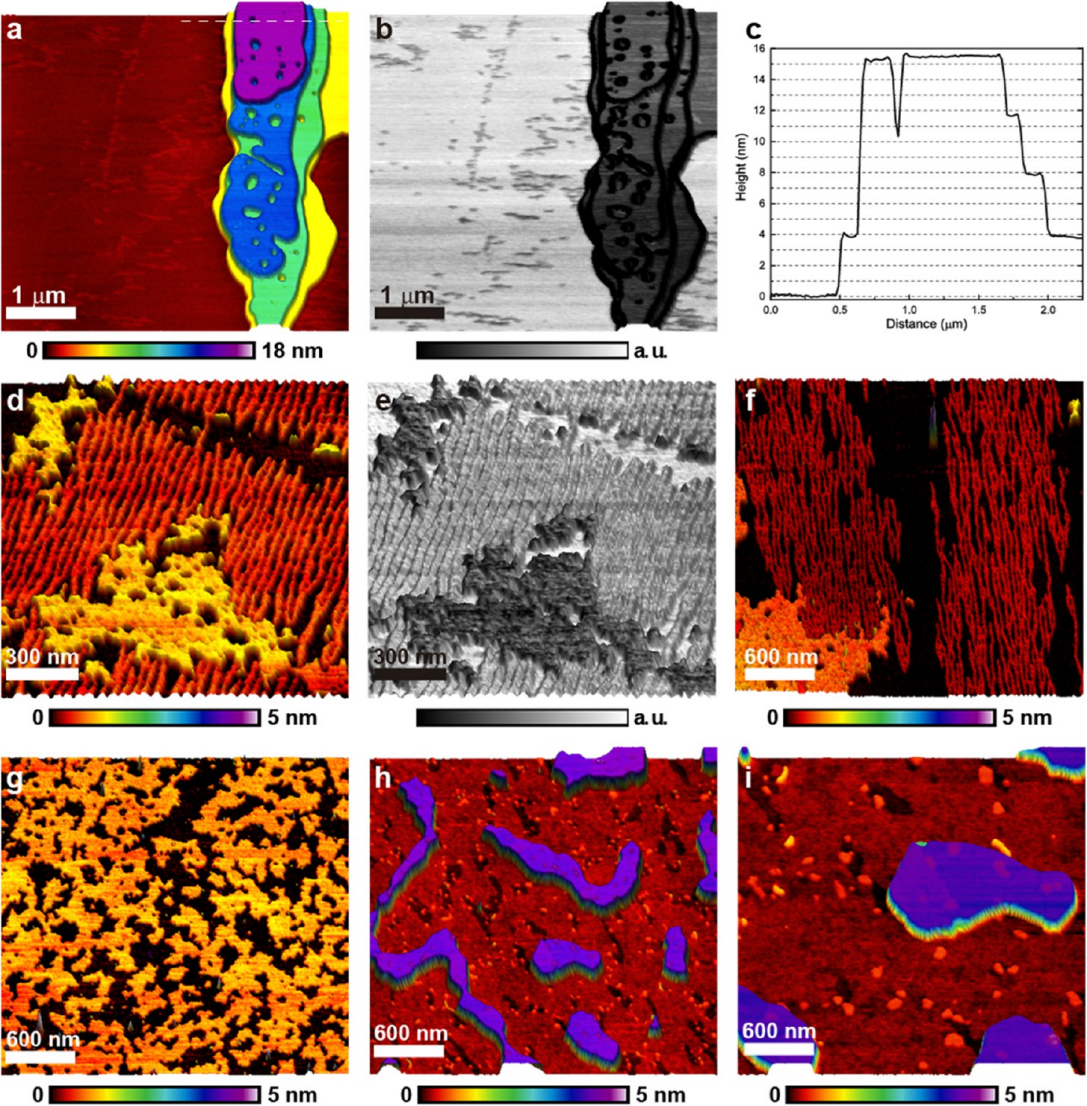
Concentrated (a–e and h–i) and intermediate (f and g) SDS solutions deposited on mica. (a) PF-QNM 3D render of a topographic image showing a multilayered SDS structure. Sample produced by spin coating using the concentrated solution. (b) Qualitative adhesion force channel image (the lighter the color, the greater the adhesion force between tip and sample) of the same region in (a), showing that the substrate is more adhesive than the layers, suggesting that the methyl groups are located on the surface of these structures while the sulfate groups are in the middle. (c) Height profile of the dashed red line in panel (a). (d) Topographic image of a region of the sample with less dense coverage and linear structures, taken 6 days after deposition. (e) Qualitative adhesion force channel image of the same region in (d), showing that the substrate is more adhesive than the thicker layers, but the linear, quasi-1D structures are more adhesive than the layers. (f) Topographic tapping mode image of a sample deposited by spin coating with the intermediate solution. The larger scan area highlights that the quasi-1D structures are long. (g) Topographic tapping mode image of another sample deposited by spin coating with the intermediate solution showing a region covered with lamellar structures. (h) Topographic tapping mode image of a sample deposited by spread coating with the concentrated solution showing a region with lamellar structures and a thinner layer covering most of the substrate. (i) Zoom-in of the field shown in the previous panel.

Images in panels d and e were taken 6 days after deposition, and panel f was taken on the same day. No evidence of temporal evolution was observed. Figure 5g shows an image of another sample deposited with the 10 mM solution, displaying regions covered with structures similar to the islands among the linear layers seen previously. These structures are approximately 1 nm thick. The linear structures were also observed on spread-coated samples. However, the longer interaction between the solution and substrate also promoted regions of fuller coverage, as seen in Figure 5, panels h and i (2 min coating time). These panels display 3D projections of topographic images of the same region (panel i is a zoomed-in region of panel h). Regions of bare substrate are scarce and appear in black. Most of the area is covered by a layer a few angstroms thick (red), similar to the linear structures seen earlier. The small islands of SDS range from 0.8 to 1.0 nm thick (similar to the islands between the linear layers). The purple layers are 3.7 nm thick. This image displays, simultaneously, most of the structures seen in other panels. The thicker (3.7 nm) structures are the layers seen in panel a (green, blue, purple) and in panels h and i (purple). Next, we have layers about 1 nm thick, seen in the middle of linear structures in panels c, d, and e, alone in panel g, and in very small areas in panels h and i. Finally, a very thin layer (around 3 Å) appears as long, linear, branched structures (panels d–f) or as islands (panels h and i, red).

We propose that the structures around 3.7 nm are bilayers, similar to those observed on talc (see the discussion about Figure 3). The thickness of the structures reported here is less than the values reported by Bernardes et al.^6,17^ and Shen^18^ and Lee^18^ (6.0 and 5.0 nm, respectively). Although both groups studied SDS aggregates on negatively charged surfaces (mica and borosilicate glass), Bernardes’ model^17^ for SDS lamellar (bilayer) structures suggests that the molecules adsorb on the surface with the CH_3_ group forming the outer surface of the layers (including directly above the substrate). Shen’s model^18^ considers the formation of a hydration layer on the substrate, followed by the adsorption of the molecules’ SO_4_^–^ group, such that the bilayers have the SO_4_^–^ groups pointing outward. Bernardes et al. studied SDS multilayers formed on mica by solvent evaporation,^17^ with the layers being hundreds of nanometers thick. In this work, we studied much thinner structures formed by spin-coating the substrate with an SDS aqueous solution. It is important to consider the interaction between the negatively charged sulfate group and the negatively charged mica surface. For layers hundreds of nanometers thick, the top layer is no longer directly influenced by the substrate. In our samples, the substrate-molecule interaction plays a major role.

In our case, low adhesion strongly indicates that the methyl group is on the surface of the structures, agreeing with the observations of Bernardes and co-workers.^17^ The thickness is compatible with the length of a single SDS molecule, suggesting that our aggregates could be a monolayer with the sulfate group close to the substrate and counterions and water molecules in between. However, this model does not explain the observed stacking of layers in Figure 5a. Bilayers of varying thickness were reported for C_n_TAB (quaternary alkyl ammonium bromides – a family of cationic surfactants) adsorbed on mica and characterized by X-ray reflectometry (XRR).^38^ The authors proposed a model with different tail arrangements: below and above the CMC, the tails are more fluid (forming thinner layers), whereas at the CMC, they are more crystalline. Bilayer thickness variations for C_12_TAB were on the order of 0.4 nm and could account for our observations of SDS, such as those in Figure 3.

Mathews^39^ and colleagues studied SDS fractal dendrites formed in eutectic solvents and reported a bilayer with a thickness of 2.0 nm, measured by X-ray diffraction. They argued that their value is lower than those previously reported for other similar SDS systems (2.5 to 2.8 nm) because the molecule could be more tilted, or its tails could form a layer with some degree of interpenetration. As mentioned earlier, hemihydrate SDS crystals are monoclinic and have a lamellar thickness of 3.1 nm,^21^ while monohydrate SDS crystals are triclinic and have a lamellar thickness of 2.9 nm.^40^ Systems of amphiphilic molecules in water can undergo transitions from a lamellar liquid crystalline phase (Lα) to α-gel and β-gel (coagel) phases, depending on temperature and other parameters,^41^ with each phase having its own lamellar thickness. Given all of this, we propose that the SDS structures observed on talc and the thicker structures on mica are bilayers with the sulfate group in the middle, accounting for the observed low adhesion force, heights, and stacking behavior. The difference in thickness could be attributed to different levels of organization of the alkyl tails (more or less crystalline—withstanding the force exerted by the AFM tip better or not), different tail tilt angles, and different hydration layer thicknesses between SDS headgroups (see Figure 1, panels c–f).

For the other two structures observed on mica – the islands around 1 nm (Figure 5g, which has the largest area covered by these) and the structure of a few angstroms, linear (Figure 5f) or not (Figure 5h) – deformation by the AFM tip is possible (as seen for the micelles on HOPG), especially if the molecules arrange in a structure with low crystallinity. The islands are less adhesive than the branched linear structures, while the bilayers are even less adhesive (Figure 5e). We propose that the islands are SDS **monolayers**, with the methyl groups residing on the outer surface, while the branched quasi-1D structures share similarities but exhibit a less dense molecular packing (see the schematic model of Figure 1b).

Given the same charge of the SDS anion and the muscovite surface, it may seem counterintuitive that a monolayer with the sulfate group closer to the substrate would form on this substrate, of all others. In water, SDS aggregates must form in a way that shields the alkyl chains from the polar water molecules, as this interaction would be energetically unfavorable. On HOPG, the alkyl chain-substrate interaction is favorable, and hemicylindrical micelles form with the molecules’ tails on top of the graphite. The talc surface, as addressed before, behaves hydrophobically, although it has water molecule binding sites. Considering this, the interaction of the talc surface with the SDS alkyl chain is neither commensurable nor favorable enough to form the epitaxial layer on which the hemimicelles grow, and molecule–molecule interactions may prevail, leading to the formation of SDS lamellar aggregates. However, the interaction between talc and a bilayer terminated by the methyl groups is more favorable than the alternative, a surface made of the highly hydrophilic sulfate groups.

On mica, however, water molecules and Na^+^ ions are strongly attracted to the surface. In this situation, the deposition of a methyl-terminated bilayer is possible, and a greater separation between this layer and the substrate may account for the observation in Figure 5a. Conversely, adsorption of individual SDS molecules on top of the hydration layer is also possible, leading to the islands and surrounding quasi-1D structures. The monolayer-like structures do not seem densely packed enough, which may inhibit the deposition of a second layer, hindering the formation of a bilayer with a sulfate outer surface. The deposition of a second layer on top of the hemimicelles was also not detected on HOPG samples. However, Figure 5h shows that a bilayer can form atop the monolayer (forming a three-layer structure). If both layers have methyl-terminated surfaces, this configuration would be more favorable than one where a methyl-terminated surface interacts with a sulfate-terminated one. The bilayer may form in the solution during the solvent removal process and stack upon the already deposited monolayer.

Sulfate groups, along with water molecules and Na^+^ ions, forming a layer without interaction with the substrate may lead to a crystalline order, as seen in SDS hydrated monoclinic and triclinic crystals, with the tails interacting via van der Waals forces and creating a more rigid structure. A thicker hydration layer, with the Na^+^ ions, could help stabilize and lead to a more crystalline arrangement. Should the sulfate groups interact with the substrate’s surface binding sites, the formation of this crystalline arrangement may be hindered by the distribution of the binding sites. Surface interactions with surfactant molecules are known to lead to the formation of surface aggregates with a different configuration than the bulk aggregates in solution.^36,42^

Before presenting the annealing test results that further support the claims made in the previous paragraphs, let us address the formation of quasi-1D SDS structures on mica. Chen and co-workers^43^ studied the assembly of peptides on (molybdenum disulfide) MoS_2_, which form 2D arrays. They showed that the building blocks form 2D films by juxtaposing rows (1D). The formation of fibers, both on surfaces and in bulk solution, can lead to the assembly of 2D and 3D structures, depending on the system’s interactions. Lenz and Witten^44^ proposed a model for the self-assembly of geometrically frustrated units, resulting in various structures, primarily fibers, and linear and branched configurations. Although SDS molecules are simpler than peptides, the formation of quasi-1D structures on surfaces may be related to the effects discussed in the two cited works. The fact that the molecule is a salt and ionized in water makes the interaction between the head groups complex, as the molecules tend to repel each other, making the interaction unfavorable. The formation of crystalline aggregates with densely packed and well-organized alkyl chains is driven by van der Waals interactions, but the repulsion of the charged head groups opposes this tendency, as does the strain from assembling misfit units. Na^+^ ions and water molecules are attracted to the headgroup, reducing the electrostatic repulsion, but Bernardes et al. reported a net negative charge^6^ on the SDS bilayers deposited on mica.

Although large aggregates of lamellar structures form on all three substrates studied here, further organization of the molecules occurs over a time scale ranging from minutes to days, suggesting the possibility of evolution into more favorable structures. In contrast, the stability of the quasi-1D structures persisted throughout the analyzed time frame (see, for example, Figure 5d). The shorter lateral size of these structures may represent a preferred organization, minimizing strain compared to both long and wide (2D) lamellar structures.

Similar features (time evolution, formation of rod-like and other quasi-1D structures) were observed in systems consisting of small alkyl chain (*n* = 8) phosphonic acid molecules and mixed molecular systems.^22,45^ Pure self-assembled structures of phosphonic acids with 14 carbon atoms in the saturated alkyl chain are much more stable. Related effects may explain the observations here. The SDS alkyl chain is not long enough to stabilize (via van der Waals forces) the structures formed, and the interactions between the charged headgroup, its neighbors, Na^+^ ions, and water may favor inverted micelles and quasi-1D lamellar structures instead of 2D lamellar structures. Geometric frustration has also been hypothesized to explain the formation of quasi-1D structures^46^ atop graphene.

Although a 2D layer structure of a few angstroms also formed (Figure 5h), the quasi-1D structures were much more common. Local fluctuations may account for these observations. The solvent removal process, achieved through spin and spread coating (and N_2_ stream to dry the sample), induces fluctuations in the local concentration of SDS in the solution and other nonequilibrium phenomena. This leads to a wide variety of morphological aggregates formed by the molecule on surfaces, compared to systems studied in equilibrium at the solid–liquid interface.

### *In Situ* Annealing Tests

We also investigated the thermal stability of the SDS structures on talc and mica by performing *in situ* annealing tests, tracking changes in the morphology of SDS aggregates as a function of temperature. We begin by discussing the results obtained on talc, followed by those on mica.

Figure 6 displays the results. The samples were deposited using the concentrated solution and spread coating (2 min), which facilitated the attainment of higher coverage and more homogeneous samples. Since during annealing the tip must be retracted from the surface and repositioned at the same region takes time and requires more scans due to thermal drift, a homogeneous sample avoided the need to keep the exact same position after each temperature variation step. Additionally, the tip may suffer damage during the test, and it must be replaced and the test restarted with a different sample. Therefore, a batch of samples was deposited under the same conditions to ensure homogeneity between samples. The annealing test was performed on this batch of samples to ensure reproducibility. Thickness measurements reported here were made using histograms, reflecting the average thickness of each structure. Representative images at each temperature are shown, selected based on consistency in annealing conditions and imaging quality, taking into account tip wear and sample variability.

**Figure 6:**
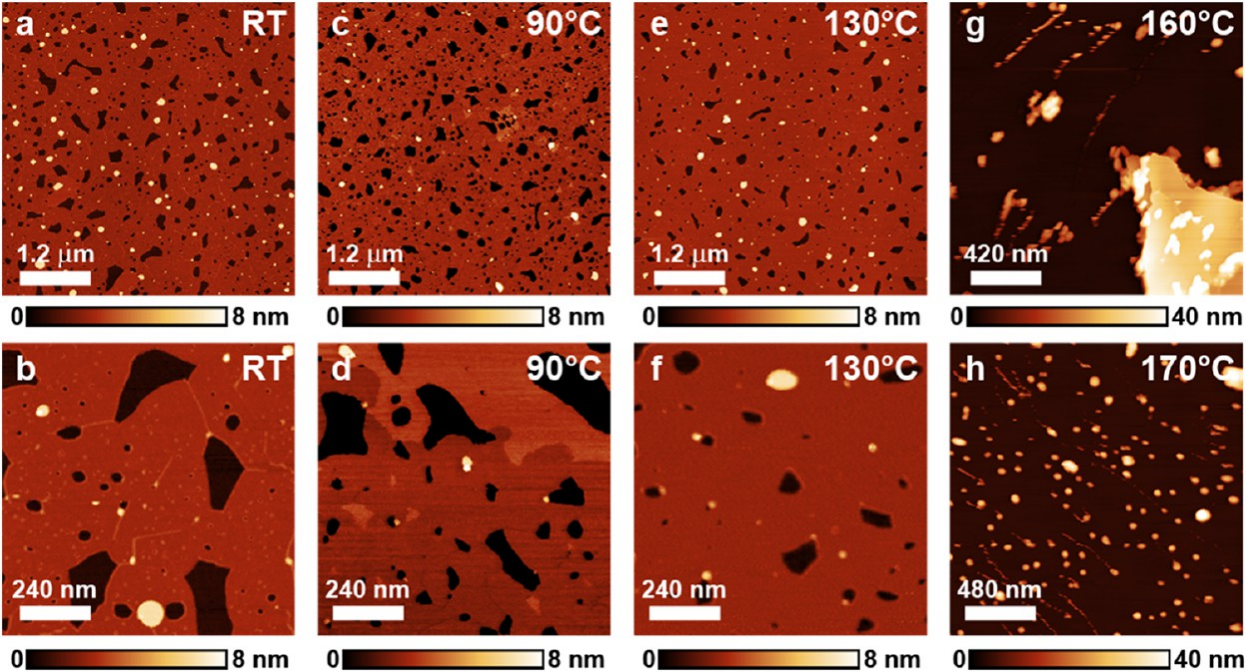
AFM tapping mode images of the concentrated 20 mM solution spread-coated on talc, *in situ* annealing test. (a) Topographic image of a region covered by lamellar SDS structures at room temperature. (b) Zoom-in of a region from the previous image. (c) Representative image at 90 °C. (d) Zoom-in of a region from the previous image. (e) Representative image at 130 °C. (f) Zoom-in of a region from the previous image. (g) Representative image at 130 °C. (h) Representative image at 170 °C.

Panels a and b show images at room temperature. The structure appears to be formed by the coalescence of lamellar structures, a few angstroms thinner (∼2.4 nm) than the previously discussed samples. A second layer is seen forming in some regions, giving rise to the lighter, rounded regions; it is similar to the one in Figure 4a. The sample was annealed to 50 °C and then to 70 °C with no significant changes. Two images from the next step, 90 °C, are displayed in Figure 6c,d. Most of the layer is 3.2 nm thick. This increase from room temperature might be due to the reorganization of the molecules or simply the evaporation of the contamination layer covering the substrate, since 90 °C would be enough to promote the evaporation of loose water molecules atop talc. The thicker regions (seen mainly in the higher magnification image of panel 6d) appeared and are 3.8 nm thick. The redistribution of water and Na^+^ ions in the hydration layer or the reorganization of the molecules in a less tilted manner could account for that.

The temperature was increased to 100, 115, and 120 °C without much change. Figures 6e,f display images taken at 130 °C. The lamellar structure tends to coalesce, with fewer defects observed. The thicker regions seen in panels c and d are no longer detected. The second layer that was present from the beginning is still visible. The lamellar thickness decreased to 2.0 nm. The SDS molecules may be gaining enough energy to transition from a dense packing (such as seen in Figure 1d) to a looser one (such as Figure 1e). Since the apparent coverage increased, it might be due to the lateral distance between the molecules increasing, leading to this decrease in thickness.

Further annealing was performed in 10 °C steps up to 170 °C. Between 150 and 160 °C, dramatic changes took place. The material aggregated, forming large agglomerates, and part of it remained loose on the substrate, being dragged by the tip while scanning (see panel g, Figure 6). At 170 °C (panel h), all lamellar organization was lost, and rounded agglomerates could be observed. After cooling down to room temperature, no further modifications took place, indicating that the change was irreversible (see Figure S5). This result is consistent with the mica sample that will be presented later and indicates that the lamellar structure is highly robust and thermally stable. Comparisons will be made after the presentation of those results.

We then turned to mica to assess whether similar structural transformations and stability patterns could be observed under thermal treatment. The same considerations regarding the experimental difficulties of these tests, discussed for the talc sample, apply here. First, Figure 7 presents the results of the annealing test for the quasi-1D structures. The test was performed on the sample displayed in Figure 5f. In the first part of the test, the same field was imaged after each temperature increase. Since each temperature step was limited to 5 °C, thermal drift between consecutive images was minimal. From panel f onward, the regions were close but not exactly the same.

**Figure 7:**
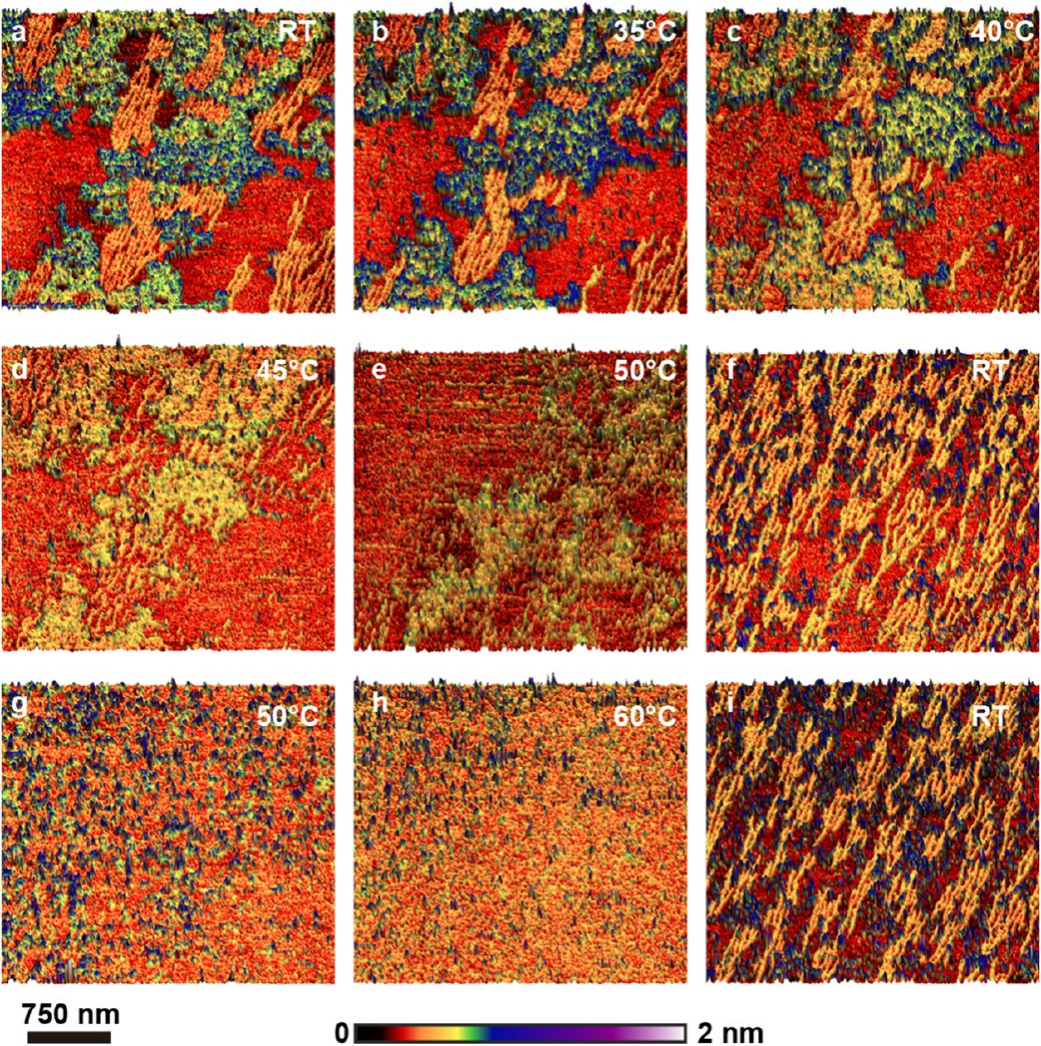
AFM tapping mode images of the intermediate 10 mM solution spin-coated on mica, in situ annealing test. (a) Topographic image of a region covered with quasi-1D SDS structures at room temperature. (b) Image of the same region at 35 °C. (c) Image of the same region at 40 °C. (d) Image of the same region at 45 °C. (e) Image of the same region at 50 °C. (f) Image near the previous region after the sample cooled down to room temperature. (g) The sample was annealed again, with a representative image at 50 °C. (h) Representative image at 60 °C. (i) Representative image at room temperature after the sample has cooled down again.

Panel 7a shows the sample at room temperature. Both the lamellar 1 nm-thick structures (green-blue) and the thin linear structures (yellow) are present. At 35 °C (panel b), the linear structures already exhibit modifications, and material (probably from them) starts to coalesce into smaller, rounded areas atop the substrate. Less reorganization is observed in the 2D structures. The deterioration of the quasi-1D structures continues at 40 °C (panel c) and 45 °C (panel d) and is almost complete at 50 °C (panel e). At 50 °C, the 2D structures also suffer more modifications. The sample was allowed to cool to room temperature, and the image in panel f was captured at 27 °C. Additional images of the same area were taken, and they all showed the reorganization of the quasi-1D structures. The lateral size of the thicker 2D islands decreased significantly. The same sample was annealed again, immediately after the room temperature images were taken, this time, to preserve the probe, directly to 50 °C. Figure 7g shows that at 50 °C, most of the organization was lost, and at 60 °C (panel h), what remained of the structures was small, difficult to scan, and very unstable. Once allowed to cool to room temperature again, the quasi-1D structures reorganized, establishing that the process is reversible (panel I).

This supports the conclusions drawn earlier. The quasi-1D structures are fragile and begin to disorganize at lower temperatures (compared to SDS on talc – Figure 6). A single layer of SDS molecules, lightly packed atop a water and Na^+^ layer, would not withstand much thermal energy due to both the mobility of the water molecules (and, eventually, evaporation) and the low crystallinity of the alkyl chain packing. The reorganization of these quasi-1D structures, together with the fact that they are much more common than a 2D monolayer as thin as they are (Figure 6h), suggests that the linear branched configuration is preferred, as would be expected for structures formed by units that induce significant strain as they aggregate.^44^

The 2D bilayers on mica exhibit very different behavior, as shown in Figure 8. A similar procedure to the one described for the talc sample was also adopted in this case. The sample was deposited via spread coating (2 min of coating time) from the concentrated solution. A region with dense coverage and piling up of bilayer structures was chosen. Fortunately, there were steps on the mica substrate in the region that facilitated keeping scanning the same area, although not exactly the same field, at each temperature increase step. Image processing for the AFM images in Figure 8 was performed to keep the bare mica regions of the higher step colored blue (3D projections).

**Figure 8:**
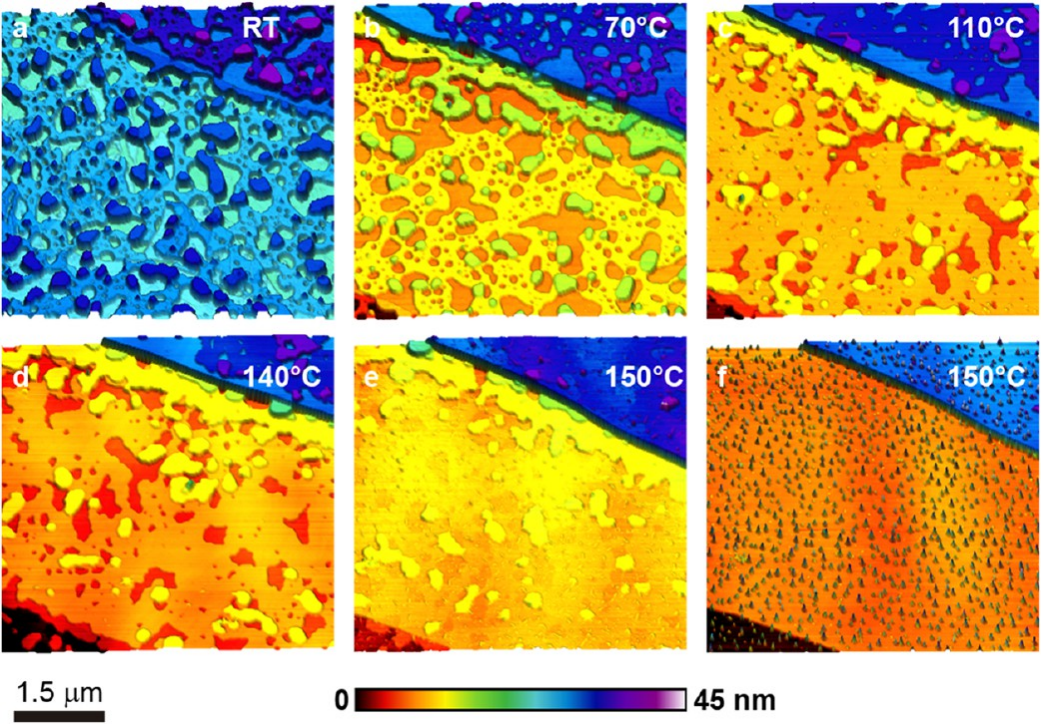
AFM tapping mode images of the concentrated 20 mM solution spread-coated on mica, in situ annealing test. (a) Topographic image of a region covered with lamellar 2D SDS structures at room temperature. A step on the substrate is visible at the top left corner, which is prominent in the subsequent panels. (b) Image of the same region at 70 °C. (c) Image of the same region at 110 °C. (d) Image of the same region at 140 °C. (e) Image of the same region at 150 °C. (f) Image of the same region at 150 °C after more time elapsed.

Figure 8a shows the sample at room temperature. Most of the substrate is covered by SDS layers, and the substrate step can be seen as a diagonal line in the top right corner. Each bilayer step is between 3.7 and 4 nm, similar to the structure in Figure 5a. A higher magnification image is shown in Figure S7. Upon annealing, the layers become mobile, as seen in the scan taken at 70 °C in Figure 8b. Substrate coverage decreases, and the mica step is now easily visible. Another step is detected at the lower right corner of the image (darker colors). No modification in bilayer thickness was observed.

At 110 °C (Figure 8c), the coverage of the first SDS layer increased, but the piling up of layers decreased significantly (an image taken at 90 °C can be seen in Figure S8a). The layer thickness decreased to approximately 3.4–3.9 nm. Up to 140 °C (Figure 8d), lateral mobility continued to alter the shape and stacking of the bilayers. At the higher mica step, the substrate appears light blue (in a few visible regions), while a ∼1 nm layer covers most of the area (dark blue). A few higher stacks (∼4 nm, purple) are also visible. Small islands of material also appeared, which can be seen in more detail in Figure S8b. Further scanning of the same area revealed that the material lost its organization and accumulated into droplets, as shown in Figure 8f. The morphology of this region remained unchanged after the sample was cooled to room temperature (see Figure S9a). The Supporting Information also includes an image taken at room temperature of a field in the same region as those already shown but not previously scanned (Figure S9b). Its morphology is similar to that in Figure 8e, suggesting that the perturbation caused by the heated probe may contribute to the final disorganization of the layers.

The behavior observed in this test follows the same trends as those observed for the sample on talc (Figure 6). The lamellar bilayers observed here vary in thickness, even among structures on the same substrate. However, they all exhibit outer surfaces with low adhesion, indicating that the ions and sulfate headgroups are located in the middle of each structure. These methyl-terminated structures should not interact strongly with either talc or mica, which explains the lateral mobility they exhibit at lower temperatures. Despite this mobility, the bilayers remain structurally stable and only lose their organization at elevated temperatures (150–160 °C). In contrast, the linear structures shown in Figure 7 are more susceptible to disruption, reinforcing the structural models proposed earlier and highlighting the superior thermal robustness of the lamellar bilayers

One possible factor contributing to the irreversible disorganization of SDS structures at high temperatures is hydrolysis, a process known to occur in aqueous SDS solutions with rates influenced by temperature, concentration, and pH.^47–49^ While the present study focuses on the solid–air interface, the presence of residual water molecules and counterions within the lamellae and at the substrate surface, as discussed previously, could allow hydrolysis. Given the temperature dependence of this reaction, it may play a role in structural changes observed above 150 °C. However, studies on related systems indicate that SDS hydrolysis typically proceeds over time scales of hours to days,^47–49^ making it unlikely to be the dominant process during our relatively short annealing experiments. Instead, visible material condensation on the AFM probe chip and tip holder following annealing suggests that desorption and significant perturbation of the system are the primary contributors to the observed structural degradation.

### Overview of Results

To conclude the Results and Discussionsection, we provide an overview that synthesizes the main findings on the self-assembled structures formed by SDS, highlighting how these morphologies vary with solution concentration, deposition method, and substrate properties, as revealed by scanning probe microscopy.

Lamellar bilayer structures (Figure 1d–f) were observed on all three substrates – HOPG, talc, and mica – using both spread and spin coating deposition methods. On HOPG and talc, these structures were detected in samples prepared from solutions both below and above the critical micelle concentration (CMC) of SDS in water. In contrast, on mica, bilayers appeared primarily in samples prepared from concentrations above the CMC.

The bilayers exhibited variable thicknesses, typically between 3.1 and 3.7 nm. This variation likely reflects differences in packing density and crystallinity, as illustrated in Figure 1 (panels d–f). Additionally, the contribution of hydration layers may influence the apparent height. On HOPG and talc, these bilayers underwent temporal evolution over periods ranging from minutes to days, indicating that intermolecular SDS interactions outweigh those with the substrate. The nonequilibrium nature of the deposition methods used—both involving forced solvent removal—further emphasizes the kinetic influence on the final structures.

Upon annealing, lamellar bilayers on talc and mica showed notable thermal stability up to at least 130 °C. However, structural mobility and rearrangements were observed at lower temperatures (70–90 °C), and irreversible layer disruption occurred at higher temperatures.

Monolayer structures (Figure 1b) were identified on mica, appearing both as 2D lamellae and as quasi-1D aggregates. These were observed in samples deposited by both methods using concentrations above the CMC. Their measured thickness—consistently below the full molecular length of SDS— indicates low crystallinity and potential deformation by the SPM tip during scanning. These structures behaved distinctly under annealing, degrading around 60 °C but reforming upon cooling. This reversibility suggests that molecular degradation does not occur, and that structural disruption is likely due to reorganization rather than breakdown.

The 2D and quasi-1D structures observed on the substrates were assigned as methyl-terminated based on two main observations. First, nanomechanical measurements revealed lower adhesion values compared to the underlying substrates, consistent with exposure of the hydrophobic alkyl chains at the surface. Second, these structures exhibited a tendency to stack or pile up, a behavior that aligns with hydrophobic interactions driving aggregation. In contrast, no such vertical stacking was observed for the hemicylindrical micelles, which are known to be sulfate-terminated, further supporting this assignment.

## Conclusions

This work investigated the self-assembly of sodium dodecyl sulfate (SDS) at the solid–air interface on three mineral substrates – HOPG (highly oriented pyrolytic graphite), talc, and mica - following solvent removal via spin and spread coating (assisted by a N_2_ stream to fully dry the samples). While previous studies have predominantly focused on SDS structures formed either at the solid–liquid interface or after complete solvent evaporation, the present study uniquely explores the intermediate regime, where the solvent is rapidly removed. Under these conditions, less SDS was deposited compared to full-drying methods, and the resulting nonequilibrium state differed from that of the solid–liquid interface, both of which strongly influenced the structures observed. The results reveal how substrate properties and solution concentration influence the formation of diverse self-assembled morphologies, as characterized by scanning probe microscopy.

Hemicylindrical micelles were detected on HOPG, consistent with earlier observations at the solid–liquid interface. Lamellar bilayer structures, generally 3.1–3.7 nm thick and methyl-terminated, were observed across all substrates. On talc, bilayers exhibited temporal evolution and spontaneous reorganization, indicating dynamic rearrangement under ambient conditions. On mica, quasi-1D and 2D structures, assigned as monolayers, were also detected; these were less thermally stable than bilayers and showed reversible behavior upon mild annealing.

Investigating SDS self-assembled structures after solvent removal posed a significant challenge due to the diversity of morphologies that could form. Conventional parameters for controlling the self-assembly process, such as solution concentration, coating method, and coating time, had limited influence on the results, and heterogeneous samples were consistently obtained. This underscores the critical role of local conditions in the formation of these systems. While we conducted a comprehensive set of experiments and proposed models for the observed structures, it is beyond the scope of this work to address all possibilities. The multitude of structures observed at the solid–air interface presents intriguing opportunities for further study.

## Supplementary Material



## Notes

Additional results for SDS-HOPG samples; description of observed lamellar structures; further discussion on temporal evolution of SDS talc samples; mica samples produced with 4 mM solution; additional images from *in situ* annealing tests (PDF)
