# Supplementary material for: Self-Assembled Sodium Dodecyl Sulfate Structures on Mineral Surfaces Following Rapid Solvent Removal
Source: Langmuir. 2025 May 20;41(21):13079–91. doi: 10.1021/acs.langmuir.5c00640 (PMC12139036; doi:10.1021/acs.langmuir.5c00640)
Supplement: Supplementary file 1 [file la5c00640_si_001.pdf]

Supporting information for:

Self-assembled sodium dodecyl sulfate structures on mineral surfaces following rapid solvent removal

Mariana C. Prado[1] and Bernardo R. A. Neves[2]*

[1]Departamento de Física, ICEB, Universidade Federal de Ouro Preto, Rua Quatro, Campus Universitário Morro do Cruzeiro, CEP 35402-136. Ouro Preto, Brazil.

[2]Departamento de Física, ICEx, Universidade Federal de Minas Gerais, Avenida Antônio Carlos, 6627, CEP 30123-970. Belo Horizonte, Brazil.

*mariana.prado@ufop.edu.br

Table of Contents:

Number of pages: 14

Number of figures: 9

A – SDS on HOPG

First, to complement the discussion in the main text about SDS (sodium dodecyl sulfate) on HOPG (highly oriented pyrolytic graphite), Figure S1 presents the results of spread coating using the dilute (4 mM) and concentrated (20 mM) solutions. Samples from the dilute solution, prepared using spread coating for 30 seconds, are shown in Figure S1a and 1b. Panel 1a shows an atomic force microscopy (AFM) image of a HOPG step covered by hemicylindrical micelles, compatible with the ones observed using the spin coating method. Panel 1b displays the FFT (fast Fourier transform) of the amplitude error channel (not shown). The periodicity of the structures measured in that image is 4.7 nm.

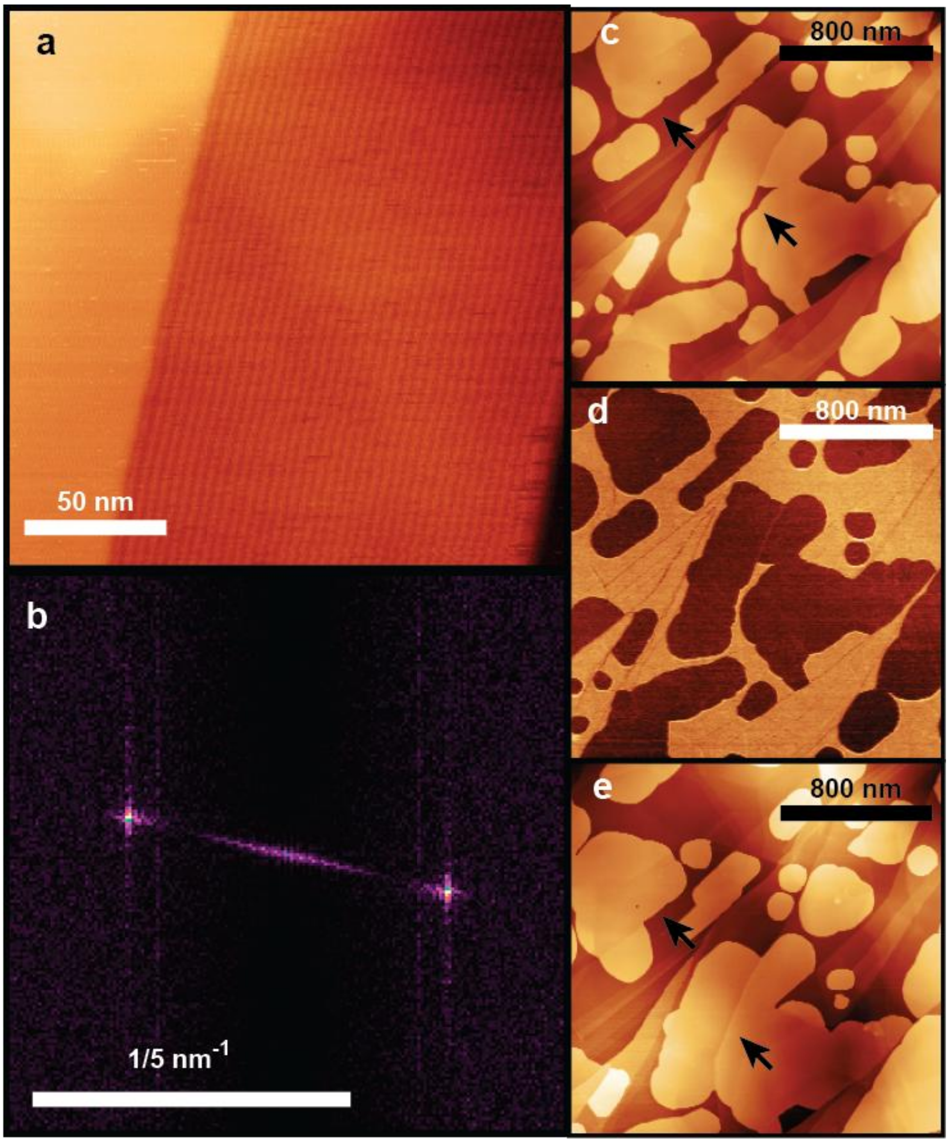

Figure S1. SDS samples produced by spread-coating dilute (a and b) and concentrated (c to e) SDS solutions on HOPG. (a) AFM topographic image of a sample spread-coated with the 4 mM (dilute) solution on HOPG, showing a step covered with SDS hemicylindrical micelles. Z scale: 3 nm. (b) FFT of the amplitude error channel (not shown) acquired simultaneously with the topographic channel displayed in (a). The periodicity measured in this image is 4.7 nm. (c) Topographic image of a sample produced by spread-coating the 20 mM (concentrated) on HOPG. Image processing was performed to enhance the visualization of the structures rather than focusing on step alignment for the HOPG. Z scale: 12 nm. (d) Qualitative adhesion force measurement. Lighter colors indicate higher adhesion force between the tip and the sample. SDS structures appear less adhesive than HOPG, suggesting that the structures are CH_3-terminated. (e) Topographic image of the same region (despite thermal drift) after 13 minutes, revealing the lateral mobility of the structures. Z scale: 12 nm. The black arrows in panels (c) and (e) guide the eye to regions that display differences between the first and second scans.

Both spin and spread coating yields hemimicelles with a periodicity smaller than usually reported for measurements made in the liquid-solid interface[1,2], for this SDS concentration in water (4 mM). That finding supports the hypothesis that local concentrations during solvent removal might be higher than the nominal solution concentration, since the periodicity is compatible with what is observed for those cases in equilibrium.

As mentioned in the main text, structures consistent with lamellar phases were also detected on HOPG samples. These structures have not been reported on samples imaged at the liquid-solid interface. Figures S1c to e display an example obtained using spread coating with the concentrated solution, imaged qualitatively using peak force quantitative nanomechanical (PF-QNM)[3] mode (see ”Experimental Section”). In fact, striped domains were not observed in samples prepared with the concentrated solution employing spread or spin coating, however, at the liquid-solid interface, literature reports describe the formation of hemicylindrical micelles at concentrations up to 100 mM[4]. This suggests that the forced removal of water hinders the formation of the epitaxial (horizontal) monolayer that serves as a template for the hemimicelles, instead favoring the deposition of lamellar structures. In Figure S1c, the layers range from 3.3 nm to 3.6 nm in thickness. Figure S1d displays the qualitative adhesion force between tip and sample, acquired simultaneously with topography data. SDS structures appear darker than the HOPG substrate, indication a lower adhesion force. That suggested that the lamellas are CH_3 terminated and the SO_4^- groups, water and counter ions are probably in the middle of the structure (similar to the model proposed by Bernardes and colleagues[5]). The polar side of the molecule is expected to result in the formation of a thicker contamination layer, which leads to greater adhesive forces due to capillarity. Considering this, the lamellae are believed to consist of SDS bilayers.

The layers exhibit lateral mobility, as evidenced by the observation that the domains coalesce over time, as shown in Figure S1c and 1e. These figures show the same region scanned twice, with an interval equal to the time required to complete one scan and begin the next, approximately 5 minutes. The black arrows guide the eye to two examples of domains that merged and changed shape during this interval. This observation suggests that the lamellar domains interact weakly with the HOPG substrate. At 20 mM, which is above the CMC (critical micelle concentration), SDS molecules are expected to form spherical micelles in water at room temperature. Our results suggest that the intermolecular interactions are sufficiently strong in this case to prevent individual molecules from forming striped hemimicelle domains. This supports the idea that a higher local concentration occurs during the deposition of samples. Furthermore, self-assembled structures at the liquid-solid interface may differ from the bulk micelles. This suggests that the former structures are truly assembled at the surface, depending on the critical aggregate concentration (CAC), which is typically much lower than the CMC[6].

Figure S2 illustrates the effect of increasing the coating time and further supports the conclusions discussed above. It shows a PF-QNM image of a 20 mM SDS solution spread coated on HOPG with a coating time of 13 minutes. Image processing was performed to highlight the features discussed next, and no quantitative information was extracted. Panel a shows the topography channel, indicating that an extended coating time increased SDS coverage, resulting in layer stacking (compare with Figure S1c). The other panels (b to d) represent the peak-force mechanical properties channels: adhesion, deformation, and LogDMT[3] (the logarithm of the elastic modulus of the sample based on the Derjagi-Muller-Toropov model). Regions that are thinner (darker) in the topography channel correspond to lighter colors in the adhesion force measurement (more adhesive), darker areas in the deformation channel (less deformable), and lighter regions in the

LogDMT channel (stiffer). This allows the identification of these regions as the HOPG substrate (due to their height, deformability, and stiffness) and shows that they are more adhesive than the surrounding layers. Even after 13 minutes of coating time full substrate coverage was not achieved. The SDS layers are, once again, less adhesive (CH_3-terminated) and, as expected, more deformable and less stiff than the substrate.

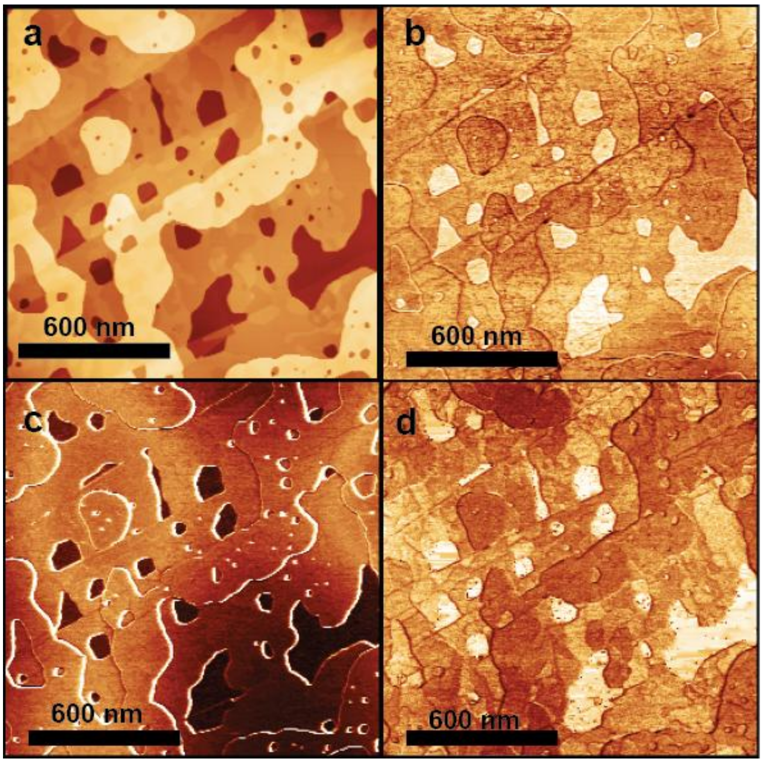

Figure S2. Lamellar SDS structures on HOPG. PF-QNM images of an SDS 20 mM solution spread-coated on HOPG (coating time: 13 minutes). (a) Topographic image. Image processing was performed to enhance the visualization of the structures rather than aligning the HOPG steps. Z scale: 15 nm. (b) Qualitative adhesion force measurement, showing that compared to the 30-second samples, SDS coverage increased with coating time. (c) Qualitative deformation measurement, showing that the SDS layers deform more (lighter) than the substrate (darker). (d) Qualitative stiffness measurement (logarithm of the elastic modulus), indicating that the substrate is stiffer (lighter) than the SDS lamellar structures.

A comprehensive exploration of SDS self-assembled structures on HOPG at the solid-air interface is beyond the scope of this work. After demonstrating that striped (hemicylindrical micelles) and lamellar structures can be formed through spread and spin coating of dilute and concentrated solutions, we now turn to other substrates.

B – SDS on talc

Let's begin by expanding the discussion in the main text about SDS on talc, starting with the results obtained from the dilute solution deposited *via* spread coating. Figure S3 displays PF-QNM images of the sample after 8 days of deposition. Panels a (topography) and b (adhesion force) show a region that appears identical to their state on the deposition day, with no signs of temporal evolution. The average thickness is 3.5 nm, as measured from a histogram plot. The adhesion channel indicates that these islands are less adhesive than the surrounding substrate. Additionally, areas of intermediate adhesion correspond to an angstrom-thick layer that is not evident in the topography channel due to the chosen z scale. These islands are very similar to the structures observed in samples produced by spin coating and employing other concentrations. The primary difference between this sample and those shown in Figures 3 and 4 of the main text is the lower coverage, which is expected given the reduced SDS concentration. This facilitates the detection of unorganized material on the substrate (the aforementioned angstrom-thick layer), which can also be observed in samples deposited with more concentrated solutions.

On the other hand, Panels c (topography) and d (adhesion force) present a high-magnification image of a region with structures **showing** signs of temporal evolution. The edges of the islands tend to transition from rounded shapes to straight lines. The larger island, located approximately in the middle of the field, has a thickness of 2.9 nm. A small, thinner region is visible at the top part of the structure, with a height difference of 0.7 nm. No considerable change in adhesion is observed, suggesting that the height difference does not originate from the surface of the island. Differences in crystalline arrangements and hydration layer thickness may account for this observation.

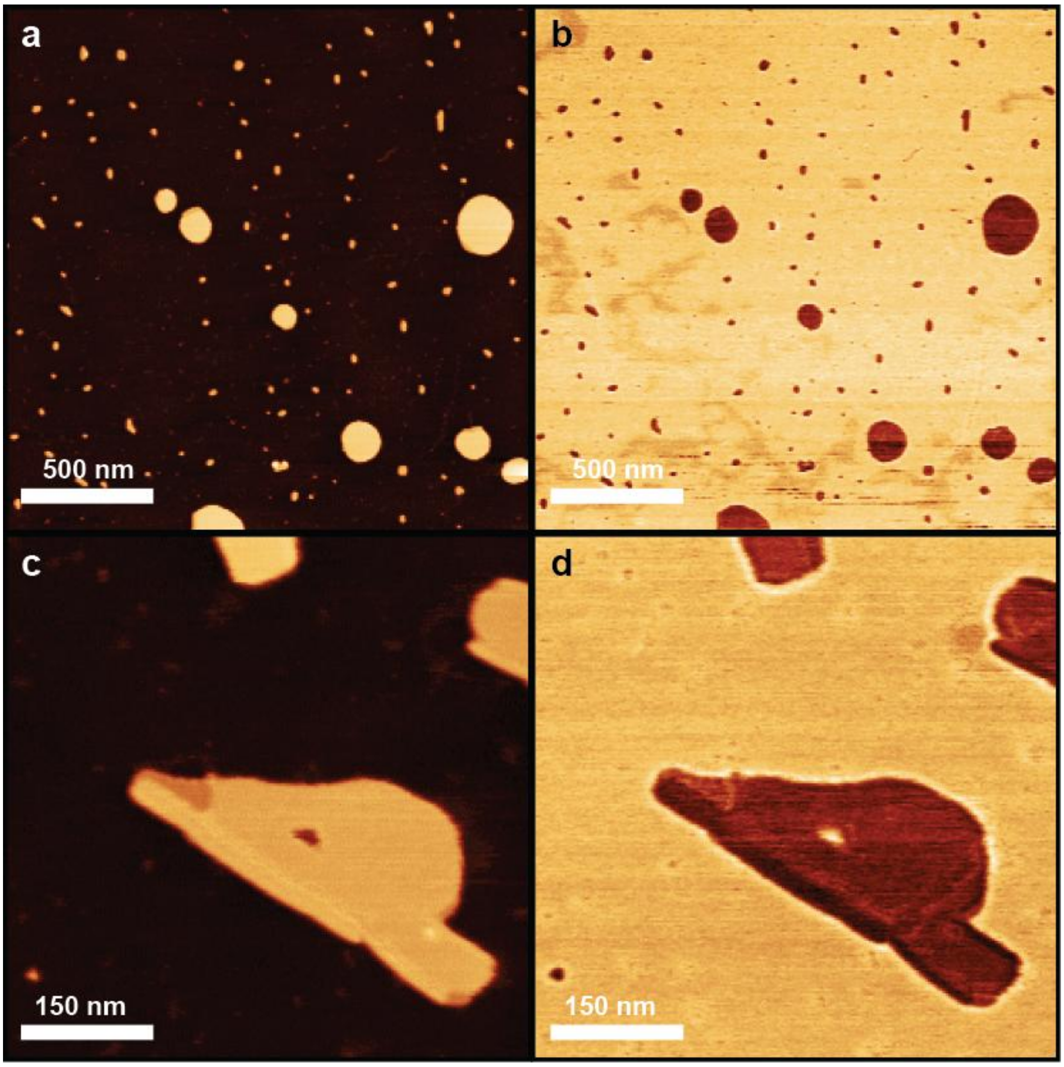

Figure S3. PF-QNM images of the dilute solution spread-coated on talc. (a) Topographic image of a sample produced by letting the solution sit on talc for 60 seconds before removing it with a pure N_2 stream. Image acquired 8 days after deposition. Z scale: 5 nm. (b) Qualitative adhesion force measurement, where lighter colors indicate higher adhesion forces between the tip and sample. SDS structures appear less adhesive. (c) Topographic image of a region containing a structure with straight borders. Z scale: 5 nm. (d) Qualitative adhesion force measurement of the same region.

For the concentrated solution, Figure S4 illustrates the effects of temporal evolution on the lamellar structures. Panels a and b present topographic and qualitative adhesion force images, respectively. The holey layer is, on average, 3.1 nm thick, similar to its thickness on the day of deposition. The layer indicated by the green arrow is 3.4 nm thick, with the structure atop it being a few angstroms thicker. The region marked by the yellow

arrow measures 3.7 nm in thickness from the substrate. Atop that structure lies a long, linear layer that is 0.4 nm thicker. This suggests that atop the more organized layer, long, linear structures measuring between 0.3 nm and 0.4 nm form and align side by side. The angle formed by the structures, indicated in blue, is approximately 63º. After 7 days, it is evident that not all layers have rearranged into these structures. However, there is a trend toward forming the thicker (3.4 nm) bilayer with geometrical edges and linear structures on top. The adhesion force image in panel b indicates that the substrate remains covered with a highly adhesive contamination layer.

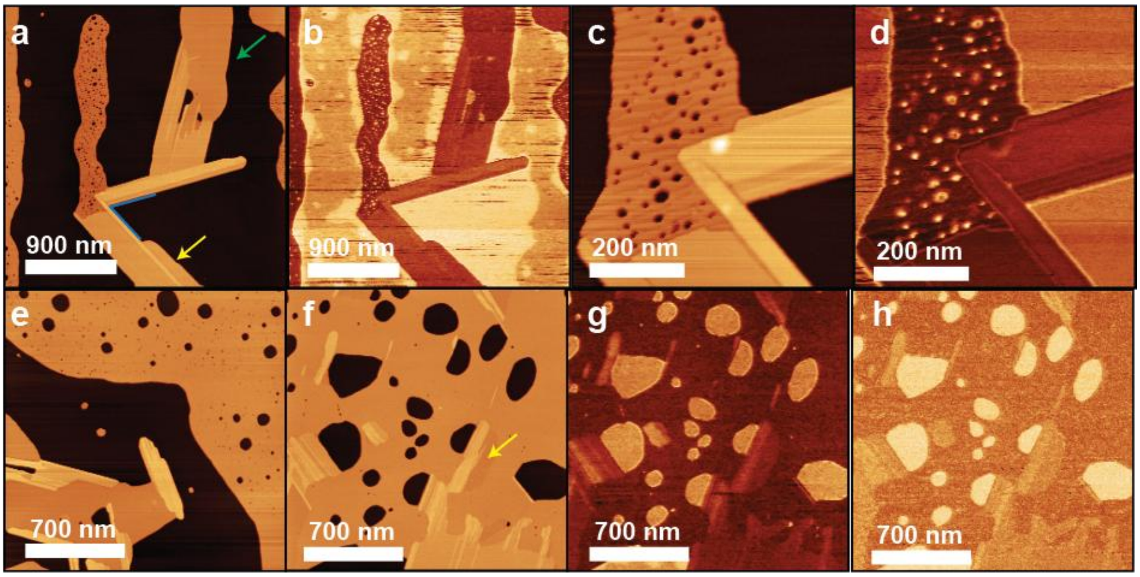

Figure S4. PF-QNM images of SDS 20 mM solution spin-coated on talc after 7 days of deposition (kept in ambient conditions). (a) Topographic image of a region covered by various types of layers. Z scale: 7 nm. (b) Qualitative adhesion force measurement, where lighter colors indicate higher adhesion forces between the tip and sample. SDS structures appear less adhesive. (c) Zoom-in view showing the rippled appearance of one of the layers in this field. Z scale: 7 nm. (d) Qualitative adhesion force measurement of the same region as panel c. (e) Topographic image of another region displaying similar types of structures. Z scale: 7 nm. (f) Topographic image of a field predominantly covered by the 3.4 nm layer. Z scale: 7 nm. (g) Qualitative adhesion force measurement of the region in panel f. (h) Qualitative stiffness measurement, where lighter colors indicate higher stiffness. SDS structures appear less stiff compared to the substrate.

Figures S4c and 4d are, respectively, topographic and adhesion force images of the region near the corner indicated by the blue lines in panel a. This layer now exhibits a

rippled appearance. The periodicity of the structures is approximately 17 nm. The adhesion force channel shows no difference in the surface of the structures compared to the other layers.

Figure S4e is a topographic image of another region of the sample, displaying the same three types of structures: a thinner, holey layer (without any indication of the ripples seen in Figure S4c), a thicker, more organized layer, and the top linear, geometrical stripes Panels f, g, and h are, respectively, topography, adhesion force, and stiffness channel images of a region covered by the thicker (3.4 nm) layer. The region indicated by the yellow arrow is approximately 2 Å below the top of the layer. On one edge of this region, linear structures approximately 4 Å higher are formed. Multiple of these structures are visible in the field, including instances of piling up. The adhesion force channel displays a slight contrast between the linear structures and the surrounding layer. Panel h is a qualitative stiffness image. It shows that the substrate is the stiffest structure in the field (lighter), as expected for a mineral substrate compared to an organic layer (similar to the case of HOPG). A small contrast is visible between the linear structures and the lamellar layer, possibly indicating that the SDS molecules are more densely packed in the linear structures.

As discussed in the main text, Cain and colleagues[7] reported on self-assembled structures of a wedge-shaped anionic surfactant (disodium-3,4,5-tris(dodecyloxy)phenylmethylphosphonate – TDPMP) on mica. They detected multilayered structures formed by periodic stripes, described as aggregates of reverse cylindrical micelles. Johnson and Nagarajan[6] proposed a series of possible aggregate structures for surfactants adsorbed on hydrophilic substrates, including monolayers topped by hemicylinders. These structures could be somewhat similar to what we observe

here, although SDS structures would be reverse micelles, as reported by Cain and co-workers[7].

C – SDS on mica

First, we present in Figure S5 an image of an SDS sample spread-coated on mica for 2 minutes using the dilute solution. Small islands of SDS are visible on the substrate, without the formation of more complex self-assembled structures.

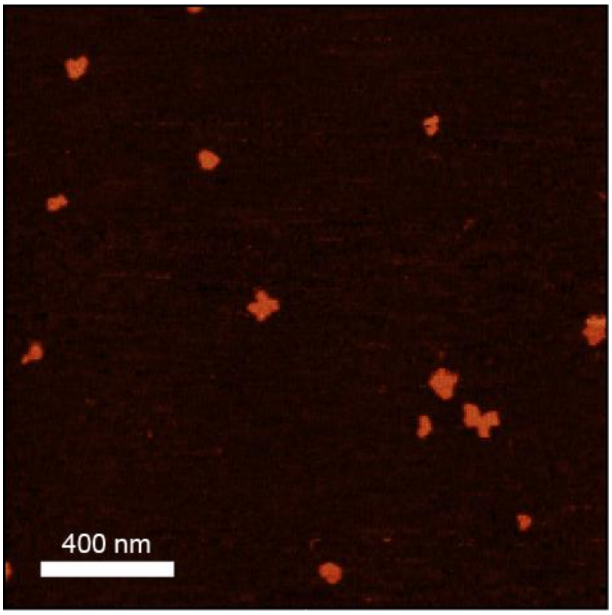

Figure S5. AFM image of an SDS dilute solution spin-coated on mica. Z scale: 2.5 nm.

D – *In situ* annealing tests

Adding to the *in situ* annealing tests reported in the main text, first we address details regarding talc samples.

Figure S6 displays an image of a sample that was subjected to a temperature of 170°C and then allowed to naturally cool down to room temperature. The image is similar to panel h of Figure 6 in the main text, providing evidence that the disorganization of the lamellar SDS structures is irreversible.

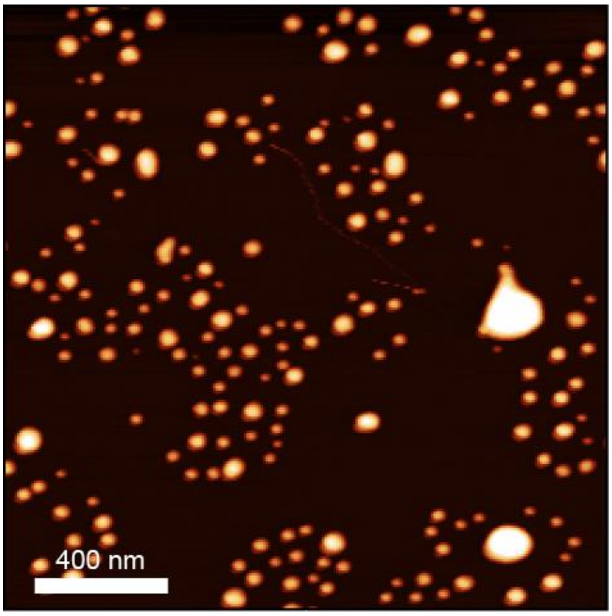

Figure S6. AFM image of an SDS concentrated solution spin-coated on talc after *in situ* annealing up to 170°C and cooling down to room temperature. Z scale 30 nm.

Finally, adding to the discussion about the thermal stability of the mica samples, Figure S7 presents a zoomed-in image of a region from Fig. 8a. The stacking of bilayers is more easily observed, and grain boundaries can be detected, indicating that the layers are formed by structures that coalesce.

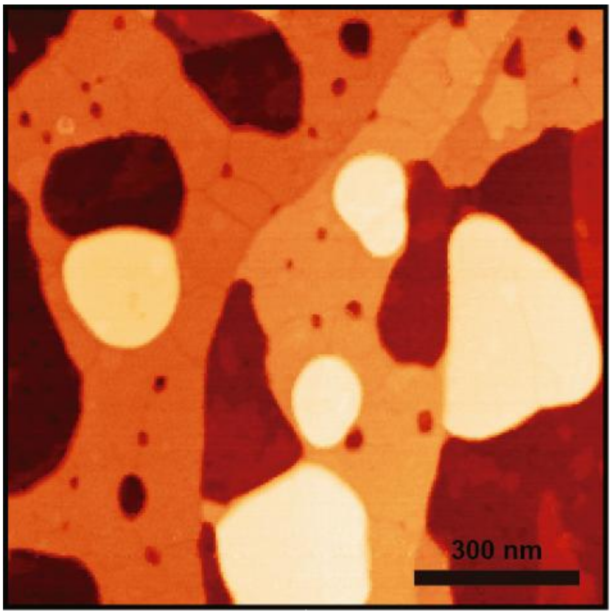

Figure S7. AFM image of an SDS concentrated solution spin-coated on mica. Zoom-in of the region shown in Figure 8a in the main text. Z scale: 13 nm.

Figure S8a presents an image taken at 90°C, which is very similar to Fig. 8b (taken at 70°C). Panel b displays a higher magnification image of the sample at 150°C. In this image, it is possible to observe the first layer, approximately 1 nm thick (dark brown), along with the remaining bilayers. Additionally, a myriad of small islands can be seen forming among the larger ones, possibly originating from material that was previously part of the bilayer lamellar stackings.

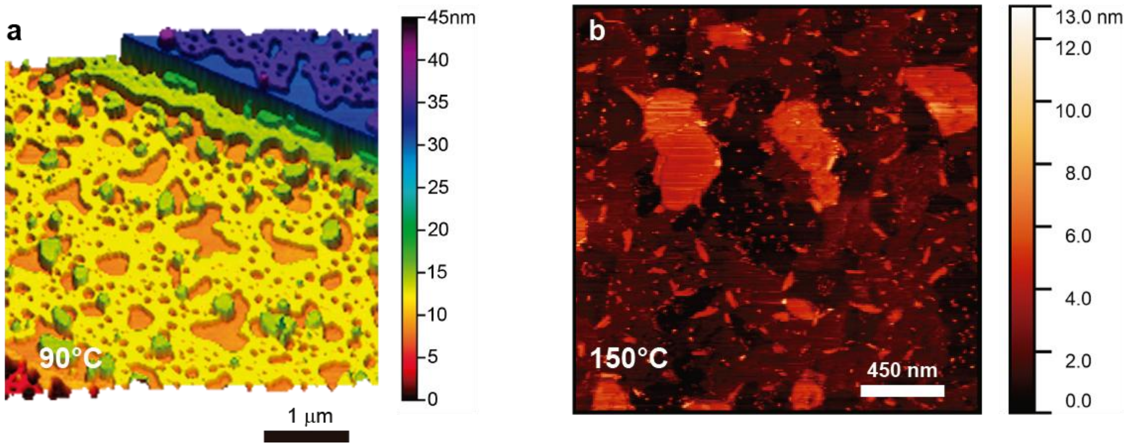

Figure S8. FM images of an SDS concentrated solution spin-coated on mica, complementing Figure 8 in the main text. (a) 3D projection of an AFM image of the sample annealed at 90°C. (b) AFM image of the sample annealed at 150°C, showing a zoom-in of the region displayed in panel f of Figure 8.

Upon cooling this sample back to room temperature, the region previously scanned (Fig. 8f) retained the same morphology (Figure S9a). Panel b presents a zoomed-in view of the region marked by the arrow. A layer of approximately ~1 nm remained covering certain areas of the substrate. Panel c shows a scan of a nearby area that was not previously scanned. It is notable that this region resembles the morphology observed at 150°C (Figure S8b), suggesting that the perturbation caused by the (also heated) probe may have contributed to the final disorganization of the layers.

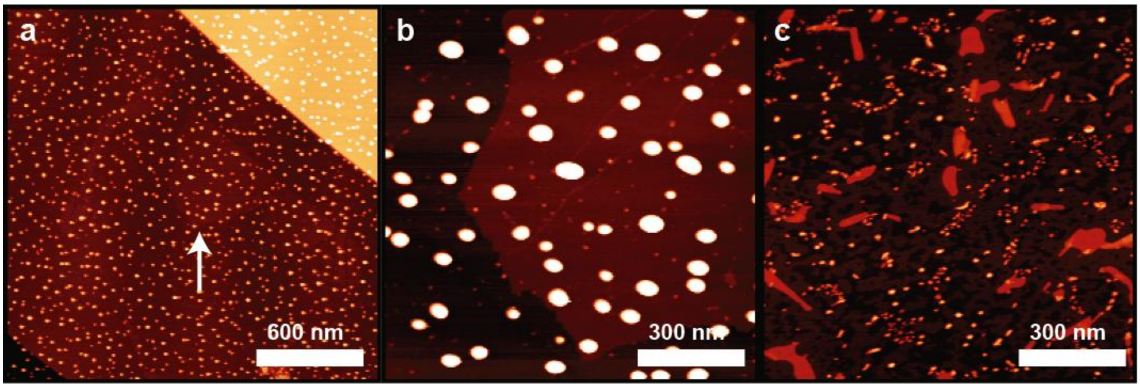

Figure S9. AFM images of an SDS concentrated solution spin-coated on mica, complementing Figure 8 in the main text after the sample was returned to room temperature. (a) AFM image of a field near the region shown in Figure 8f, displaying a thin layer covering the substrate in some areas, as indicated by the arrow. Z scale: 45 nm. (b) Zoom-in of the previous image, showing the structures in more detail. The corner of the layer indicated by the arrow in (a) is located at the bottom left of the image. Z scale: 13 nm. (c) AFM image of another field, close to the regions in the previous images but not previously scanned, similar to the one in Figure S8b. Z scale: 13 nm.

SI References

(1) Wanless, E. J.; Ducker, W. A. Organization of Sodium Dodecyl Sulfate at the Graphite−Solution Interface. *J. Phys. Chem.* **1996**, *100* (8), 3207–3214.

(2) Wanless, E. J.; Ducker, W. A. Weak Influence of Divalent Ions on Anionic Surfactant Surface-Aggregation. *Langmuir* **1997**, *13* (6), 1463–1474.

(3) Dokukin, M. E.; Sokolov, I. Quantitative Mapping of the Elastic Modulus of Soft Materials with HarmoniX and PeakForce QNM AFM Modes. *Langmuir* **2012**, *28* (46), 16060–16071.

(4) Umeda, K.; Kobayashi, K.; Yamada, H. Nanomechanics of Self-Assembled Surfactants Revealed by Frequency-Modulation Atomic Force Microscopy. *Nanoscale* **2022**, *14* (12), 4626–4634.

(5) Bernardes, J. S.; Rezende, C. A.; Galembeck, F. Morphology and Self-Arraying of SDS and DTAB Dried on Mica Surface. *Langmuir* **2010**, *26* (11), 7824–7832.

(6) Johnson, R. A.; Nagarajan, R. Modeling Self-Assembly of Surfactants at Solid–Liquid Interfaces. II. Hydrophilic Surfaces. *Colloids Surf. Physicochem. Eng. Asp.* **2000**, *167* (1–2), 21–30.

(7) Cain, N.; Van Bogaert, J.; Gin, D. L.; Hammond, S. R.; Schwartz, D. K. Self-Organization of a Wedge-Shaped Surfactant in Monolayers and Multilayers. *Langmuir* **2007**, *23* (2), 482–487.
